# Correction: *A qualitative study exploring the social and environmental context of recently-acquired HIV infection among men who have sex with men in South-East England*

**DOI:** 10.1136/bmjopen-2017-016494corr1

**Published:** 2017-12-22

Gourlay A, Fox J, Gafos M, *et al*. A qualitative study exploring the social and environmental context of recently acquired HIV infection among men who have sex with men in South-East England. *BMJ Open* 2017;**7**:e016494. doi: 10.1136/bmjopen-2017-016494.

The current email address of corresponding author Annabelle Gourlay is annabelle.gourlay@lshtm.ac.uk.

Mitzy Gafos is also affiliated to the Department of Global Health and Development, Faculty of Public Health and Policy, London School of Hygiene and Tropical Medicine, London.

