# Supplementary material for: A qualitative study exploring the social and environmental context of recently acquired HIV infection among men who have sex with men in South-East England
Source: BMJ Open. 2017 Aug 28;7(8):e016494. doi: 10.1136/bmjopen-2017-016494 (PMC5629694; doi:10.1136/bmjopen-2017-016494)
Supplement: Supplementary file 1 [file bmjopen-2017-016494supp001.pdf]

Social factors related to HIV acquisition
Interview discussion guide

Introduction

- Thank for agreeing to participate
- Introduce self and organisation in collaboration with clinics
- Recap study objectives
- Remind participant they can stop the interview at any time or decline to answer
- Double check consent for recording the interview

Ice-breaker & background

Can you tell me a little bit about yourself and your background?

- How old are you?
- Where are you from originally (born there)? A city or a rural area? Where do you live now? Who are you living with? How long have you been living there? If moved, when? From where? Why?
- Do you have family? Who? (Parents/ siblings – older or younger) Where are they living?
- What education have you received? University/higher education?
- Are you employed (or student)? What? Where? Since when?
- Are you in a relationship? Since when? Where is your partner from? If not in a relationship, previously in a relationship? When did this end? Relationships with men only, or men and women?
- Have you told anyone that you are gay or bisexual? When did you come out? How old were you then?

Migration

[If not originally from this city]

You said you moved to London/Brighton in yyyy. If you think back to that time, how were you feeling and how did you find this transition?

- Moved alone or with someone else/ others?
- Sources of support?
- Any challenges?

Circumstances around HIV infection

Could you tell me about your life in the last year or so, just before you discovered you were HIV positive?

- Monogamous/exclusive sexual relationship(s)?
- Or more casual sexual partners/ dating? Nature and number of partners? Preferred casual partners, or seeking long-term relationship?
- Where and how you met? Social activities? Clubs, bars, sex clubs, saunas, cruising (public environment)? Any change in habits?
- Recent travel (sex) abroad? (e.g. Amsterdam, Paris, Berlin)
- Internet, dating apps, other social media? Any change in use?
- Experienced sexual violence (in last year, or before, e.g. forced you to have sex)? Or other physical abuse (e.g. he hit you)? Shouting or threatening you (emotional abuse)? Any other frightening behaviour?
- Friends?
- How were you feeling emotionally (psychological well-being)? Self-confidence? What triggered any change?
- Health?
- Work?
- Financial situation?
- Home and family?
- Moving/re-location?
- Lifestyle?
- Drug or alcohol use? If yes, how did you get involved in/ what triggered this? Why?
- Any other challenges faced during this time?
- Negative reactions towards your sexuality/being gay? (Stigma (homophobia))

Do you know when you might have caught HIV? How? From whom?

- HIV-status and nature of sexual partner(s)?
- Sexual behaviour (condom use, breakage, receptive/insertive partner, ejaculation?)
- Were you (ever) forced into sex?
- To what extent did you see this [e.g. unprotected sex] as a risk to your sexual health? Why? (Risk perception and attitudes to unprotected sex)
- Did you try to manage this risk [e.g. sex without condom]? How? Any challenges? Ever discussed this with anyone – partners, friends, health or other support services? (Risk management)
- How were you feeling at this time?
- Drug (e.g. poppers, ecstasy) or alcohol use before or during sex?
- How did you (or other HIV-positive peers) become involved in [drug use/ high risk practices/ gay social scene]?
- What (other) factors do you think contributed (to risk behaviour and getting infected)? (Probe for social factors above) How?
- Why do you think you got HIV *now*? What factors or events triggered this?

Overcoming challenges and staying HIV-negative

You mentioned […] were difficulties you faced at this time. How might any of these difficulties/events have been overcome?

- Support services? What exactly? By whom? How would this have helped?

What would have helped you to stay HIV negative?

Previous STI/HIV testing patterns and counselling

Can you tell me about any contact you had with health services for STI testing *before* you were diagnosed with HIV?

- How many times did you get tested for HIV?
- How did you react to these (negative) results? Effect on sexual behaviour?
- Diagnosed with other STIs? Which? When?
- Experiences with using these services?
- Perceptions of the counselling given (including drug use counselling). What advice given? Helpful or not? How easy to understand the advice or put it into practice? Timing of advice (started receiving in time/ too late?)
- Any other advice or support (clinic-based or other, e.g. social groups, internet-based) you received, or wish you had received?

Initial perceptions of HIV care and support services

Some people can find it difficult to continue attending clinics or to take treatment in the long-term. Based on your initial experiences of the HIV services you have received and your personal life at the moment, do you anticipate any difficulties with being able to continue attending?

- What difficulties?
- Disclosure (planned/already? How did that go?) Experienced/anticipated negative reactions (stigma)?
- Probe for any of the social or psychological factors discussed above
- Attended/ experiences or perceptions of other non-clinic support services?
- What would help you to continue attending/ follow clinical advice?

END OF INTERVIEW

- Thank participant for their time
- Reassure confidentiality of the discussion
- Provide information on relevant support services
