# Supplementary material for: A qualitative study exploring the social and environmental context of recently acquired HIV infection among men who have sex with men in South-East England
Source: BMJ Open. 2017 Aug 28;7(8):e016494. doi: 10.1136/bmjopen-2017-016494 (PMC5629694; doi:10.1136/bmjopen-2017-016494)
Supplement: Author's manuscript [file bmjopen-2017-016494.draft_revisions.pdf]

BMJ Open is committed to open peer review. As part of this commitment we make the peer review history of every article we publish publicly available.

When an article is published we post the peer reviewers' comments and the authors' responses online. We also post the versions of the paper that were used during peer review. These are the versions that the peer review comments apply to.

The versions of the paper that follow are the versions that were submitted during the peer review process. They are not the versions of record or the final published versions. They should not be cited or distributed as the published version of this manuscript.

BMJ Open is an open access journal and the full, final, typeset and author-corrected version of record of the manuscript is available on our site with no access controls, subscription charges or pay-per-view fees (http://bmjopen.bmj.com).

If you have any questions on BMJ Open's open peer review process please email editorial.bmjopen@bmj.com

# The psycho-social and environmental context of recently-acquired HIV infection among men who have sex with men in South-East England

| | |
|---|---|
| Journal: | *BMJ Open* |
| Manuscript ID | bmjopen-2017-016494 |
| Article Type: | Research |
| Date Submitted by the Author: | 22-Feb-2017 |
| Complete List of Authors: | Gourlay, Annabelle; University College London, Department of Infection and Population Health<br>Fox, Julie; King's College London, Guy's and St Thomas' NHS Trust<br>Gafos, Mitzy ; University College London, MRC Clinical Trials Unit<br>Fidler, Sarah; Imperial College London, Department of Medicine<br>Nwokolo, Nneka; Chelsea and Westminster Hospital NHS Foundation Trust, 56 Dean Street, Department of HIV/GUM<br>Clarke, Amanda; Brighton and Sussex University Hospital , Elton John Centre<br>Gilson, Richard; University College London, Department of Infection and Population Health<br>Orkin, Chloe; Barts Health NHS Trust, Ambrose King Centre, Royal London Hospital<br>Collins, Simon; HIV i-Base<br>Porter, Kholoud; University College London, Department of Infection and Population Health<br>Hart, Graham; University College London, Department of Infection & Population Health |
| <b>Primary Subject Heading</b>: | HIV/AIDS |
| Secondary Subject Heading: | Qualitative research, Sexual health, Mental health, Epidemiology |
| Keywords: | HIV & AIDS < INFECTIOUS DISEASES, Epidemiology < INFECTIOUS DISEASES, Public health < INFECTIOUS DISEASES, MENTAL HEALTH, QUALITATIVE RESEARCH |

# The psycho-social and environmental context of recently-acquired HIV infection among men who have sex with men in South-East England

Annabelle Gourlay[1,2], Julie Fox[3], Mitzy Gafos[1], Sarah Fidler[4], Nneka Nwokolo[5], Amanda Clarke[6], Richard Gilson[1], Chloe Orkin[7], Simon Collins[8], Kholoud Porter[1,2] and Graham Hart[1]

**Affiliations**:
1 Faculty of Population Health Sciences, University College London, London, UK
2 National Institute for Health Research Health Protection Research Unit in Blood Borne and Sexually Transmitted Infections, UK
3 Guys and St Thomas' NHS Trust/ King's College London, London, UK
4 Imperial College London, London, UK
5 56 Dean Street, Department of HIV/GUM, Chelsea and Westminster Hospital NHS Foundation Trust, London, UK
6 Elton John Centre, Royal Sussex County Hospital, Brighton and Sussex University Hospital, Brighton, UK
7 Ambrose King Centre, Royal London Hospital, Barts Health NHS Trust, London, UK
8 HIV i-Base, London, UK

**Correspondence to**: Dr Annabelle Gourlay, Department of Infection and Population Health, Faculty of Population Health Sciences, University College London, Mortimer Market Centre, off Capper Street, London WC1E 6JB. Telephone: +44 (0)20 7679 7611. Email: a.gourlay@ucl.ac.uk

Abstract word count (283) Manuscript word count (5982) Tables (1) Figures (2)

**Suggested running head**: Social context of recent HIV infections among MSM in South-East England



For peer review only - http://bmjopen.bmj.com/site/about/guidelines.xhtml

## Abstract

**Objectives**: A key UK public health priority is to reduce HIV incidence among gay and other men who have sex with men (MSM). This study aimed to explore the social and environmental context in which new HIV infections occurred among MSM in London and Brighton in 2015.

**Design**: A qualitative study, comprising in-depth interviews, was carried out as a sub-study to the UK Register of HIV Seroconverters cohort: an observational cohort of individuals whose date of HIV seroconversion was well-estimated. An inductive thematic analysis was conducted in NVivo, guided by a socio-ecological framework.

**Setting**: Participants were recruited from 6 HIV clinics in London and Brighton. Fieldwork was conducted between January and April 2015.

**Participants**: All MSM eligible for the UK Register Seroconverter cohort (an HIV-positive antibody test result within 12 months of their last documented HIV-negative test, or other laboratory evidence of HIV seroconversion) diagnosed within the past 12 months and aged ≥18 were eligible for the qualitative sub-study. 21 MSM participated, aged 22 – 61 years and predominantly white.

**Results**: A complex interplay of factors, operating at different levels, influenced risk behaviours and HIV acquisition. Participants saw risk as multifactorial but the relative importance of factors varied for each person. Individual psycho-social factors, including personal history, recent life stressors and mental health, enhanced vulnerability towards higher risk situations, while features of the social environment, such as chemsex and social media, and prevalent community beliefs regarding treatment and HIV normalisation, encouraged risk-taking.

**Conclusions**: Recently-acquired HIV infection among MSM reflects a complex web of factors operating at different levels. These findings point to the need for multi-level interventions to reduce the risk of HIV acquisition among high risk MSM in the UK and similar settings.




For peer review only - http://bmjopen.bmj.com/site/about/guidelines.xhtml

For peer review only

## Strengths and limitations of this study

- This study provides important insights into the social and environmental contexts for recent HIV infections among gay and other men who have sex with men (MSM) in the United Kingdom, where reducing HIV incidence remains a public health priority.
- A unique feature of our study, carried out as a sub-study to the UK Register of HIV Seroconverters cohort, was the recruitment of participants known to have recently acquired HIV infection, maximising their ability to recall contextual information.
- Although rich data were collected and many respondents voluntarily discussed sensitive personal issues, social desirability bias may still have been present.
- All participants were recruited from London/ Brighton and most were white, possibly limiting the generalisability of findings to other areas or ethnic groups, although our findings are likely to apply to similar UK or international cities with large populations of MSM.

**Keywords**

HIV; United Kingdom; MSM; recent HIV infection; sexual behaviour; mental health; chemsex

For peer review only

## Background

Gay men and other men who have sex with men (MSM) accounted for more than half of individuals newly-diagnosed with HIV in the UK in 2015 (1), with evidence of a rising trend in HIV incidence in recent years and continued high rates of transmission (2-4). Understanding the context in which new infections occur in this key population therefore remains a priority (5).

Qualitative and quantitative research conducted in the early 2000s identified behavioural factors associated with the risk of HIV acquisition among MSM in the UK, including condomless sex and an increasing number of partners, as well as the use of poppers (nitrite inhalants) and psychological factors such as depression (6, 7). However, psycho-social factors were not explored or reported in detail and their importance over a decade later remains unclear. More recent analyses using data from the PROUD trial identified ≥2 condomless sex partners in the last 90 days and having a bacterial rectal sexually transmitted infection (STI) in the previous year as the strongest risk factors for HIV acquisition (8). However, qualitative insights into behavioural or social factors associated with HIV acquisition were not available.

Important changes have occurred over the last decade in HIV prevention and the social environment in which MSM interact. New medical interventions, such as the use of antiretroviral treatment (ART) as prevention (9), pre-exposure prophylaxis (PrEP) (4) and the availability of HIV self-testing kits are likely to influence perceptions of risk, risk behaviours and risk of HIV acquisition. For example, sexual behaviours might be influenced by the growing awareness that having an undetectable viral load (VL) dramatically reduces the risk of HIV transmission (10). The context of sex between men has also changed dramatically, with the widespread availability of sexual networking through websites or mobile phone-based applications ('apps') and greater availability of psychoactive drugs used for 'chemsex' (specifically mephedrone, γ-hydroxybutrate/γ-butyrolactone (GHB/GBL) or crystal



For peer review only - http://bmjopen.bmj.com/site/about/guidelines.xhtml

methamphetamine, taken intentionally to enhance sex). Evidence is emerging of high levels of engagement in these behaviours (11-13) with almost one in three HIV-positive MSM participating in a UK-based HIV clinic survey and 44% of HIV-negative MSM enrolled in the PROUD PrEP study reporting chemsex in the previous year (12, 14). However, the specific role of chemsex or social media and sexual networking in the acquisition of HIV or other STIs is less clear.

Relatively little is known about the social context of and behavioural responses to medical interventions, chemsex drugs or sexual networking media. Research is also lacking on how these factors, importantly, interrelate or interact with individual-level psycho-social factors, such as mental health, to influence HIV risk. Baral et al argued that, although epidemiological studies have traditionally focussed on individual-level risk factors, it is essential to capture data that characterise multiple levels of HIV risk, including higher order social and structural-level risks (15). Levels of HIV risk and complex associations of factors at different levels can be appropriately investigated and depicted using socio-ecological frameworks, in which individuals and individual-level risks are conceptualised as part of the wider community and policy environment (Figure 1) (15, 16).

Through interviews with MSM who had recently acquired HIV infection, we set out to: i) explore the social and environmental contexts in which HIV acquisition occurred among MSM in London and Brighton in 2015; ii) investigate how these contexts influenced HIV risk; and iii) inform the design of public health interventions to help MSM reduce the risk of HIV acquisition.



For peer review only - http://bmjopen.bmj.com/site/about/guidelines.xhtml

## Methods

### *The UK Register cohort study*

We conducted a qualitative sub-study to the UK Register of HIV Seroconverters: an observational cohort of individuals whose date of HIV seroconversion was well-estimated (17). Eligible individuals were ≥16 years of age and had an HIV-positive antibody test result within 12 months of their last documented HIV-negative test, or other laboratory evidence of HIV seroconversion. Dates of seroconversion were estimated based on the latter, or the mid-point between last HIV-negative and first HIV-positive test result.

### *Selection and recruitment of participants for interview*

The qualitative study was undertaken within the National Institute for Health Research Health Protection Research Unit (NIHR HPRU) in Blood Borne and Sexually Transmitted Infections (http://bbsti.hpru.nihr.ac.uk/) (18). All MSM eligible for the UK Register, diagnosed within the past 12 months, aged ≥18 years, and recruited from 6 centres in London and Brighton, South-East England, were eligible for the qualitative sub-study. Centres were selected based on levels of recruitment to the cohort study and location, aiming to include areas with differing patient demographics. We restricted recruitment to these areas for logistical reasons and in view of their importance in the UK HIV epidemic, with over half of new HIV diagnoses among MSM in the UK made in London (2) and large MSM populations in both cities. HIV doctors undertook recruitment, explaining the study aims and providing information sheets. Participants who gave their written consent to participate were contacted by an independent researcher (AG), or by a research nurse, as preferred, to schedule the interview.

Recruitment occurred between January-March 2015 until a minimum of 20 participants had been recruited and data saturation was reached on most themes. Thirty-six men were invited, five



For peer review only - http://bmjopen.bmj.com/site/about/guidelines.xhtml

declined and six expressed interest but did not return to give consent. Of 25 who consented, one could not be contacted and three were unavailable for interview in the time-frame. Two men were subsequently excluded from the cohort as their last negative test result could not be verified, but were included in the qualitative study.

***Data collection***

In-depth interviews were conducted between February and April 2015 by a female researcher (AG). Interviews took place in private rooms at each clinic and lasted 1-1.5 hours. Participants were advised they could decline to answer questions or stop the interview at any time.

The interviews followed a discussion guide (Supplement 1) including personal background, moving to London/Brighton and experiences of this transition, if applicable, life in recent years before HIV diagnosis, relationships, and perceptions of the circumstances at the time of HIV infection. The guide was adapted during fieldwork to accommodate emerging themes.

Interviews were audio-recorded using a password-protected digital recorder and summary notes were written after each interview. Recordings were transcribed verbatim and audio-files destroyed thereafter. Transcripts were password-protected and stored on a secure computer network with restricted permissions. Transcripts were labelled with codes (e.g. 'participant_A_date') and data reported anonymously.

***Data analysis***

Summary notes informed preliminary analyses. A thematic analysis was then conducted on the interview transcripts using NVivo 11 software. Data were indexed and categorised to construct an analytic thematic framework. An inductive approach was used, further aided by topics on the discussion guide. A sub-sample (>10%) of transcripts was verified by a second researcher (MG) by



For peer review only - http://bmjopen.bmj.com/site/about/guidelines.xhtml

For peer review only

constructing thematic maps and comparing themes, which showed a high level of consistency. New categories identified at this stage were added and some categories refined. The revised framework was then re-applied to all transcripts. Data were subsequently summarised using thematic matrices (19) in Microsoft Excel to enable a comparison of themes within and across cases (interviews). The research team discussed themes at several stages of the fieldwork and analysis. A socio-ecological model was used to organise themes and data presentation (Figure 1) (15, 16).

***Ethics approvals***

The qualitative study was approved by West Midlands, South Birmingham National Research Ethics Service, as an amendment to the UK Register protocol (reference 04/Q207155). Participants gave informed signed consent, including for the audio-recording of interviews.

For peer review only



For peer review only - http://bmjopen.bmj.com/site/about/guidelines.xhtml

## Results

Interviews were completed with 21 participants, within 5 months, on average, of their HIV diagnosis and 6 months from their estimated date of HIV seroconversion. Participants were aged between 22 and 61 with a median age of 38, were mostly white, well educated, and employed (Table 1).

We first describe the psycho-social factors, experienced by the majority of participants, at the individual and interpersonal-level that increased vulnerability to risk, including HIV acquisition. We then present themes at the community and structural-level, including the social risk environment and community perceptions of HIV, which enabled risk-taking. The relative importance of factors at each level and the way they came together to influence risk are illustrated in the final section and in Figure 1.

### *Individual and interpersonal*

#### *Early influences on self*

Experiences during childhood, within the family, school and the broader socio-cultural environment, had early and long-lasting impacts on mental health, drug use, and support structures. While some respondents reported strong relationships with family members, the majority described dysfunctional or superficial relationships with their parent(s), characterised by a lack of love or attention, abandonment, arguments, physical or verbal abuse, or parents/ siblings with alcohol or mental health issues. Many participants described how this led to low self-esteem, lack of confidence, insecurity, unhappiness, anxiety and depression.



For peer review only - http://bmjopen.bmj.com/site/about/guidelines.xhtml

> *My father was...an alcoholic and he used to beat my mother and me...That may have had some impact on how destructive one is, and the fact I never had any unconditional love is something that I have struggled with in adulthood.* (Aged 40-49)

Family alcoholism occasionally influenced participants' own drug or alcohol habits.

> *I had a very heavy weed habit for a number of years and it'd be mum downstairs drunk and me upstairs stoned.* (Aged 20-29)

Early separation from family contributed to loneliness and early drug use for some participants, as described by one man whose parents moved abroad and sent him to boarding school from a young age:

> *I think probably in the past I have been quite depressed, perhaps even lonely, being on my own here in the UK.* (Aged 20-29)

School-based bullying was recalled by several participants, with similar psychological repercussions.

> *I: And were you able to identify any reasons behind that [anxiety]?*
> *R: Yeah, lots and lots: mum, dad, school...I was far too shy a kid for boarding school. I'd get squished in the corner.* (Aged 20-29)

A few men were raised in homophobic or highly religious cultures, mostly abroad and/or in rural settings, which shaped self-perceptions regarding sexual identity.



For peer review only - http://bmjopen.bmj.com/site/about/guidelines.xhtml

For peer review only

> *I grew up in a hostile environment...a small village [abroad], and being gay is not what parents wish for their kids...If you are gay it's quite difficult for yourself.* (Aged 40-49)

***Disclosure of sexual orientation***

Most participants had disclosed their sexual orientation as teenagers or young adults. A few had initially resisted or concealed their sexual identity and discussed the psychological implications of self-denial.

> *I was trying to change myself into a straight man...It didn't quite work out...I was basically on my own. My family didn't want to see me again...So the signs of depression were already there.* (Aged 40-49)

Several self-identified and came out as gay in their mid-twenties or thirties. For some, early concealment and/or late disclosure were associated with resentment regarding missed opportunities, prompting a desire for sexual exploration.

> *Growing up in an environment where you are getting to know yourself quite late, you get to...thinking about experiences and seeing other sexual stuff that you might not have needed to think about before because you are a bit behind...You know, am I missing out on stuff?* (Aged 40-49)

Participants had commonly experienced negative reactions to their disclosure. Several described 'traumatic' responses, including physical violence and rejection, which led to feelings of guilt, or depression.

For peer review only



For peer review only - http://bmjopen.bmj.com/site/about/guidelines.xhtml

*It [coming out] was quite traumatic at the time. My mum has never knowingly under-reacted to anything.* (Aged 40-49)

***Recent life stressors***

Recent stressful events experienced prior to HIV diagnosis caused psychological distress for many participants. These encompassed severe illnesses or deaths of relatives, relationship break-ups, partner/ sexual violence, loss of friendships, and bouts of physical ill-health.

*I: Can you identify a particular trigger for that [depression]?*

*R: I think it was a consequence of events...It was...falling in love with someone who I couldn't be with...splitting up with my long term partner.* (Aged 40-49)

A number reported financial or work stress, including harassment, as key life stressors.

*About 2013, that's when everything started to get sour at work...a lot of stress, being really unhappy...relying on drinking.* (Aged 30-39)

Loneliness and isolation were common outcomes, associated with fragmentation of support networks.

*There was a lot of work stress...and then friends were leaving London...and being single and not meeting anyone.* (Aged 30-39)

A few middle-aged men described a 'forty-something crisis', unfulfilled ambitions, or wavering self-worth.



For peer review only - http://bmjopen.bmj.com/site/about/guidelines.xhtml

*I mean it probably was the perfect storm you know, they [drugs] got me at a time when you know, mid-forties when I wasn't that secure, there were a few issues, I was looking for fun...it was an escape and it seemed at the time that it was...enjoyable.* (Aged 50-59)

***Accumulation of factors***

Several individuals were exposed to multiple psycho-social risk factors from a young age which accumulated gradually.

*We have to go back to my childhood...I think everything adds a weight and it's been add and add and add to a point where I lost control...I decided to let myself go.* (Aged 40-49)

In other cases, or additionally, a series of de-stabilising, traumatic events occurred suddenly.

*The year before last it was like every other week something was happening or somebody was dying and my mum had a stroke, my dad had a heart attack, [partner's] dad was involved in a really bad car crash and nearly died and...then his auntie was diagnosed with motor neurone and it was like "anything else?"...It was just so much.* (Aged 30-39)

In both scenarios, individuals became overwhelmed, unable to cope, lost control or experienced psychological trauma, including low self-worth, loss of purpose, depression and suicidal thoughts.

*There was so much going on...I was probably overwhelmed, you know. I wasn't in a stable place, I wasn't in a stable relationship, I wasn't stable financially. I had just suffered some pretty serious losses in terms of my immediate family. It was kind of all over the place really.* (Aged 40-49)



For peer review only - http://bmjopen.bmj.com/site/about/guidelines.xhtml

***Impacts on risk behaviour***

Psychological impacts sometimes directly affected sexual behaviour, for example, sex sought for escapism or self-validation. One man, who said he never "*felt nurtured*" by his parents explained:

> *I always need validation from people...and that manifests itself in a sexual context.* (Aged 40-49)

Loneliness and a desire for intimacy also prompted condomless sex. For example, one man who described himself as previously "*very strict with sex practices*" said:

> *It was probably because of the breakup of my relationship I was just feeling a need to be close to people, and that often came out in me choosing to have sex, unsafe without a condom.* (Aged 30-39)

Some individuals deliberately put themselves at risk, for example having condomless sex, including with HIV-positive partners, to self-harm or re-gain control, but did not indicate this was a recent change in their behaviour.

> *A lot of the difficulties I have...were about feeling controlled or not in control…so I knew what the risks were...but it was my choice, my decision [to have condomless sex with known HIV-positive partners].* (Aged 30-39)

Changes in attitudes to risk were also brought about by emotional trauma. A few participants described re-evaluating the potential costs of unsafe sex and the risk of HIV-infection, relative to other more important life events, or justifying their risk-taking during transient periods of psychological distress. Others cared so little for themselves that getting HIV seemed inconsequential.



For peer review only - http://bmjopen.bmj.com/site/about/guidelines.xhtml

*I didn't value my life...Because so much had happened and I'd been through so much in the past 3, 4, 5 years with...break ups and losing everything and emotional things and deaths and God knows what else, it almost becomes a bit 'all my life has just been so crap anyway what's the point, do I really care if I get it [HIV] anyway?'.* (Aged 30-39)

***Community and structural***

***Social risk environment and norms***

Participants were attracted to London and Brighton for the open-minded culture, freedom, and social opportunities offered, although most had lived in these urban areas for a number of years. Almost all had socialised or met sex partners at saunas, clubs, private sex parties, 'chill-outs' (after-parties, typically involving drugs and/or group sex), or cruising grounds. For a few city workers, the intensity of partying was stimulated by challenging and high-paced work environments. Temptations of the scene were hard to resist for some men, while physical proximity to venues increased social opportunities and often exacerbated high-risk behaviours.

*It would feel exciting and just so 'out there'...I'd feel like I'd walked into Brazil but I was still in London, it was a very colourful scene...There was a period...when I went quite crazy...every weekend*. (Aged 20-29)

Many participants talked about the excesses of partying on the scene and roughly half talked about their experiences of weekend-long binges of drugs and sex.



For peer review only - http://bmjopen.bmj.com/site/about/guidelines.xhtml

For peer review only

*You go to Vauxhall [district of South London with a high-density of gay venues] on a Friday night as a gay man and don't come home until five days later. I think there is so much to tempt young people these days, I include myself in that.* (Aged 20-29)

Social media, including geo-locational sexual networking apps, also provided convenient access to multiple sexual partners for many participants, regardless of age. Social media were often used deliberately in pursuit of sex and/or drugs and widened sexual networks including with HIV-positive men.

*The introduction of gay apps just makes the sex so much more promiscuous...I think people get a rush from it. I probably did at the time. These apps...just make it so much more convenient. They had these code words for 'come round to ours and do this'...'have this drug'...'chill-outs'...It all escalates.* (Aged 20-29)

The gay scene was described as 'fun', 'vibrant' and 'exciting', but also 'reckless', 'dangerous' and 'abusive'. Several men had experienced physical or sexual violence at saunas or nightclubs, while cruising, or through meeting people via social media.

*Around that time there were two rapes on me...one happened in...a night club down the road...I went under on G...and was attacked.* (Aged 20-29)

Several participants recalled changes in the gay scene, particularly the culture of drug use and sex. Changes were described in drug choices (e.g. mephedrone, GBL/GHB, crystal methamphetamine), context (chemsex), modes of administration (normalisation of injecting), and increasing availability of drugs at lower costs.



For peer review only - http://bmjopen.bmj.com/site/about/guidelines.xhtml

> *Drugs have changed...there are more choices...GHB, mephedrone...which I was quite scared of in the beginning...but then it's normalised in the gay scene and you just tend to do what other people do. Same thing goes for injecting. I mean these days it's not seen as so scary.* (Aged 40-49)

A few other participants discussed the role of social norms and networks, as well as the pursuit of pleasure, as reasons for getting into chemsex:

> *There's more drug taking in my social circle than I realised. I was quite naive about it...I never felt the need for them [other recreational drugs], although I did occasionally take them, but it was the sex thing...I think it was discovering how good the sex was...on drugs in terms of the duration and intensity.* (Aged 50-59)

Chemsex enhanced and extended sex, reportedly impaired judgement, decreased risk perceptions, and moved boundaries.

> *I wasn't particularly stoned but you lose certain inhibitions and...I know there was a guy having sex with me, I was bottom, and I knew he had a condom to start off. But...I'm not quite sure he had a condom after.* (Aged 50-59)

***Community perceptions and experiences of HIV***

Respondents, particularly middle-aged or older men, described shifting perceptions about HIV. Some felt that the absence of stark media campaigns and deaths from AIDS associated with the 1980s-1990s had re-shaped attitudes towards HIV, reducing fears and risk perceptions.



For peer review only - http://bmjopen.bmj.com/site/about/guidelines.xhtml

For peer review only

*Have you seen the old HIV/AIDS adverts? When you see that on TV...it's really scary, so there was more of a fear factor...Now there's no fear factor.* (Aged 50-59)

HIV was now frequently likened to having asthma or diabetes, was less concerning than hepatitis C, and was no longer viewed as a death sentence.

*I think in London it's almost got to the point where people are not that concerned about it anymore. It's not looked at as a death sentence. I remember reading an article by a doctor, which I know a lot of gay people seem to have read...that he would rather have HIV than diabetes.* (Aged 30-39)

Living a normal, healthy life with HIV was often attributed to the availability of ART and good medical care. Believing that HIV is a manageable condition, reduced fears of HIV, and knowing healthy HIV-positive men led to complacency, with some men admitting their general denial or disregard of risks.

*It's [HIV] perceived as sort of asthma now...everyone knows somebody positive now and knows that they're fit and healthy and they take a few pills a day...That's a huge factor in why so few people use protection anymore...because it has become a treatable illness...I think it changed everyone's risk calculations, because even if the worst did happen, it was no longer the worst.* (Aged 20-29)

Several participants said they balanced risks by testing regularly for HIV and other STIs, and/or that repeated negative results had encouraged further risk-taking.

*I get into the way of thinking, 'oh it doesn't matter, you're going to die anyway one day...If*



For peer review only - http://bmjopen.bmj.com/site/about/guidelines.xhtml

*you get sick you can take the pills' and it all becomes a bit blasé and a bit reckless. So it's taking a risk and then going to the clinic and finding out you're all clear.* (Aged 50-53)

Participants commonly associated an undetectable viral load, and occasionally ART, with lower risk of HIV transmission, a few specifically recalling scientific evidence. Some participants, typically younger men, made conscious choices to engage in condomless sex, including with HIV-positive (usually undetectable) men, influenced by their awareness of transmission risks and/or availability of ART, beliefs that HIV was no longer the worst outcome, or was inevitable considering the 'high prevalence of HIV in London'.

*So we weren't always safe...I've read a lot...I think there was a French one [study] which was undetectable partner, negative partner, X number of however many tens of thousands of interactions, no transmissions. So, background reading kind of made me feel more comfortable with it [condomless sex with HIV-positive ex-partner].* (Aged 20-29)

Another participant, who said he had always had condomless sex, highlighted how sex with HIV-positive undetectable men was sometimes perceived as less risky than with HIV-negative men:

*I was going to try and minimise the risks...and so I slept mostly with undetectable guys. I tried to avoid negative guys like the plague...Everyone told me and what the research told me too was that they were the ones...the only ones who were going to possibly infect.* (Aged 20-29)

A few participants mentioned PrEP spontaneously and had asked for it at their sexual health clinics, but were not able to access it, while several had taken post-exposure prophylaxis (PEP) or discussed common perceptions of PEP as a backup option. Generally, many respondents thought the



For peer review only - http://bmjopen.bmj.com/site/about/guidelines.xhtml

availability of ART, PrEP, PEP, or HIV tests, '*the undetectable factor*', changing discourses around HIV, or declining stigma had created a sense of apathy towards risks and susceptibility to HIV in the gay community. One man explained:

> *It's almost like they've been cured, 'Oh yeah, I'm HIV-positive but I'm undetectable'...which lessens the risk*. (Aged 20-29)

For peer review only

***Combined effects at the interface of individual and community levels on risk***

A minority of participants thought they could pinpoint their source of HIV infection, although several indicated the point of infection was not important to them and mentioned they did not like to dwell on this or blame anyone. Most men thought they had contracted HIV from a casual sexual encounter at a club, sauna or sex party, or from someone they had met via social media. A few suspected their source of infection was a more regular partner, while a few cases of violence, for example sexual coercion or being purposefully overdosed by someone, were possibly related to HIV infection. Some participants discussed changes over time in their sexual risk-taking, for example increasing frequency of sex or beginning to have condomless sex, which were occasionally rationalised in terms of recent stressors. However, a greater number of men did not think their sexual behaviour had changed, and some were surprised they were not infected sooner.

Few participants thought that their HIV infection was the result of a single factor. More generally, participants thought that combinations of factors at both individual and community/structural levels contributed to risk behaviours and HIV infection. Within the combination of factors that contributed to HIV acquisition (Figure 1), the relative importance of factors from each level differed for each person.



For peer review only - http://bmjopen.bmj.com/site/about/guidelines.xhtml

A few participants specifically attributed their risk of HIV to early psycho-social issues as well as the social environment. For example, one participant felt his self-harming sexual behaviours stemmed from childhood and the violent relationship with his mother, but also highlighted the role of the "*abusive*" environment, including gay saunas, and normalisation of drug use:

> *I think with the sex, I think it's...environment, especially in South London. The increase of risk sex, chemsex, is becoming an epidemic, in my opinion. You hear of so many young gay men now who are positive...and through this lifestyle. It's very hedonistic, really nasty...I think, subsequently, living in South London has made me get HIV.* (Aged 20-29)

Some explained how psycho-social issues resulted in their elevated use of drugs or sexual networking apps, which in turn widened their exposure to contexts for higher risk sex such as sex parties and chemsex.

> *I think the drugs make you want to do things like that [sex/ apps]. You know…being unhappy at work, the incredible amount of stress I went through, all of that combined made me feel a bit lonely as well...So I started to rely a bit on Grindr.* (Aged 30-39)

Psychological issues and drug use were often mentioned in combination. For example, one participant identified the important factors in his HIV infection as:

> *The drugs...but also depression because I didn't care about taking risks...I gave up*. (Aged 40-49)

Some participants who experienced psycho-social issues also identified ART and changing



For peer review only - http://bmjopen.bmj.com/site/about/guidelines.xhtml

perceptions of HIV as factors that had consciously, or sub-consciously, influenced their risk behaviours. Risk-benefit decisions regarding sexual practices were altered, influencing decisions to engage in condomless sex, including with HIV-positive men, or imbuing a more general disregard of risks.

> *When we were young adults, the fear of God was put into us. If you got it [HIV] you died...and suddenly you get to a point now where it is manageable...you could live a normal life...and you are thinking 'Okay, I live in a city with very high prevalence of this disease. I am at a crossroads in my life in terms of what I want to experience sexually'...I think the trauma that I have gone through has changed what I perceive as real risk in life...There are far more important things...So it was a combination of all those different aspects, I had actually come to the conclusion that if I did become HIV-positive it wouldn't be a big event in my life.* (Aged 40-49)

The environment also enabled sexual exploration, particularly for those who had initially contained their sexuality, for example due to socio-cultural influences in their childhood. For a few participants, moving to London and fragmenting support networks had coincided with coming out, exposing them to new social and sexual norms including sex parties, group sex and chemsex.

> *I was like a kid in a sweet shop. I wanted to experience everything, but I didn't necessarily have the rhetoric [support] around me to keep me safe.* (Aged 30-39)

Other participants thought that the social environment played a more substantial role than psychological factors in the build up to their diagnosis. For example, one man emphasised that recent stress, such as his father's ill-health, had less bearing on his "*dangerous lifestyle*" and HIV infection than the draws of the social scene:



For peer review only - http://bmjopen.bmj.com/site/about/guidelines.xhtml

*Probably they [stressors] were to a lesser extent than 'I want to go out and have fun because I'm getting old and I'm not going to be able to do it in ten years' time', and that pull was probably stronger than any of the negative stuff about my life*. (Aged 50-59)

Some felt strongly that increasing use of sexual networking apps had played an important part in their own infection, or rising HIV infections in general, or that certain apps promoted promiscuity and irresponsibility.

*I know for me that [apps] was the major factor. If the internet didn't exist, if these social sexual networking sites didn't exist, if I wanted sex, I would be forced up to Soho or whatever.* (Aged 40-49)

However, others re-iterated that it was difficult to distinguish the primary factor amidst a range of social environmental influences.

*The sex and the drugs and the apps all intertwined simultaneously and I can't really say which one led to the other.* (Aged 20-29)

Figure 2 gives worked examples of three contrasting cases, using the socio-ecological model, demonstrating the combined effects of influences at each level and differences in their relative importance.

For peer review only

## Discussion

This qualitative study among recently-infected MSM revealed a complex interplay of factors at the level of individuals and their community that influence risk behaviours and HIV acquisition. The relative importance of factors at each level varied for each person, with individual psycho-social factors enhancing vulnerability towards sexual risk situations, and features of the social risk environment and prevalent community beliefs encouraging risk-taking. A synergy of factors at the interface of these levels was ultimately important in affecting risk. Our findings reveal the broader context around new HIV infections in this population.

Psycho-social issues including early experiences of physical abuse, social rejection, isolation, drug use, anxiety or depression were common and heightened vulnerability to risk. Mental health conditions including major depression, anxiety disorders, suicidal tendencies, psychological distress and low self-esteem have been consistently reported at higher levels in MSM versus heterosexual men in high-income settings (20, 21). Several studies, predominantly American and including MSM populations, have also linked psycho-social issues such as depression, partner violence and childhood sexual abuse with condomless sex or risk of HIV acquisition (6, 22-25).

Notably, we found that a considerable number of participants had experienced multiple psycho-social issues over their life-course and/or a sudden series of traumatic events in the few years preceding their HIV diagnosis. These severely damaged their psychological well-being and appear important in placing some individuals at particularly high risk of engaging in unsafe sex or acquiring HIV. The magnified effect of experiencing multiple psycho-social issues is consistent with 'syndemic' theory, proposed initially to describe concurrent and mutually exacerbating epidemics, including poverty, racism, violence, substance use and HIV, in ethnic minority populations in the United States of America (USA) (26). This concept was later extended to urban gay men, with depression, partner



For peer review only - http://bmjopen.bmj.com/site/about/guidelines.xhtml

For peer review only

violence, suicidality, substance abuse and HIV identified as inter-related reinforcing epidemics (21, 24). Another USA-based longitudinal study has since provided compelling evidence that the accumulation of psycho-social conditions predicts HIV-related risk behaviours and HIV seroconversion among MSM (23).

Environments that normalise risk-taking, which in itself is a natural part of sexual discovery, can increase the likelihood of HIV acquisition, particularly for men whose individual-level experiences and other circumstances reduce their prioritising of sexual health. Chemsex was frequently reported in our sample and facilitated exposure to potential risks, for example through impaired judgement. This concurs with the findings of another qualitative study conducted in South London, which demonstrated that chemsex can carry more risk than drug-free sex, typically through longer duration and a greater number of partners (27). Underlying psycho-social factors, early-onset or more recent stressors, were occasionally related to engagement in chemsex in our study, although social norms sometimes appeared to be stronger influencing factors.

Sexual networking apps were commonly an accessory to drugs and sex, making it easy to meet partners and connect with networks where risk of HIV transmission might be higher. Our findings complement those of a recent cross-sectional study among young MSM in Britain and Ireland, suggesting that higher odds of high risk condomless sex were associated with, though not necessarily caused by, longer use of these technologies (13). However, our results highlight the difficulty in disentangling the role of sexual networking apps from other social environmental influences, including chemsex and other drug use, or underlying psycho-social influences.

Influenced by structural factors including the availability of ART, HIV was widely perceived among the men interviewed to be preventable and manageable, shaping attitudes to risk and behavioural norms that, in turn, played a role in HIV acquisition. Some men had developed risk-reduction



For peer review only - http://bmjopen.bmj.com/site/about/guidelines.xhtml

strategies accordingly, while reduced perceptions of risk also led more generally to complacency in terms of sexual practices. Findings to date on this topic have been mixed (28-32), with causality difficult to infer. However, our findings support those of more recent studies (10, 33), suggesting that optimism about HIV/treatment is playing an increasingly important role in sexual risk-taking.

The main strength of our study lies in the richness of data collected, covering a broad range of recurrent themes. Furthermore, interviews took place relatively recently after infection when recall was likely to be good. Nonetheless, details of participants' likely source of infection and related events may, naturally, be limited in accuracy. Many respondents disclosed sensitive personal issues, although we cannot rule out the possibility of social desirability bias. Participants were exclusively recruited from London/ Brighton and most were white, so the generalisability of findings to MSM in other areas, or to Black, Asian and other ethnic minorities (BAME), may be limited. However, we focussed on a key population of public health importance in the UK (2). It is also likely that our findings are applicable to MSM in other large cities, in the UK or similar international contexts, with sizeable MSM populations.

Our findings highlight the need for expanded access to packages of interventions that address exposures at multiple levels, if we are to limit new infections in this population. Examples already exist of multi-level or multi-faceted interventions (34) which, moving forwards, must be prioritised, particularly in the context of diminishing funding for sexual health and fragmentation of health service commissioning. Key components of a multi-level intervention would include, ideally, at the individual-level: improved clinical assessments to identify high-risk HIV-negative MSM who may benefit from enhanced support, including tailored support for young MSM, given the prominence of psycho-social issues, some of which emerged during adolescence; providing timely and flexible support, for example on-site in STI clinics and available immediately after testing, or via virtual web-based technologies; and outreach activities to link vulnerable MSM to peer or community-based



For peer review only - http://bmjopen.bmj.com/site/about/guidelines.xhtml

support groups, considering the deleterious effects of isolation and lack of social support. To address community-level risk factors, programmes should incorporate the provision of further information and education about chemsex, ideally including on-site advice in STI clinics. Training of health professionals on the social context around chemsex and sexual norms is essential, to enable culturally-competent discussions with MSM about their sexual behaviour, including engagement in chemsex, their risk-reduction strategies, and potential risks. In addition, sexual networking platforms are well-placed to deliver targeted interventions, including chemsex awareness and counselling options, while peer-led initiatives could assist in addressing complacency associated with shifting perceptions of HIV and ART, for example, by raising awareness of the social and psychological implications of an HIV diagnosis. At the structural level, our findings support policies that include access to PrEP, in combination with adherence support and promotion of frequent STI testing, for periods in which MSM find themselves at elevated risk due to compounded psycho-social life stressors.

In this paper we have shown that recently-acquired HIV infection among MSM reflects a complex web of factors operating at different levels. Individuals who experienced multiple stressors, gradually over the life-course or more suddenly, were especially vulnerable to HIV and being drawn into sexual risk situations, while the social environment created a context which enabled risk of HIV infection. While individuals were exposed to different sets of contributing circumstances, the interface of factors at each level was important in influencing risk behaviours and HIV acquisition. The circumstances surrounding HIV acquisition are complex and therefore require multi-level interventions that address individual and interpersonal psycho-social, community and structural level risk factors.



For peer review only - http://bmjopen.bmj.com/site/about/guidelines.xhtml

## Acknowledgements and funding

We would like to thank all the participants who took part in the interviews, as well as the clinic staff who assisted with recruitment. This report is independent research by the National Institute for Health Research. The research was funded by the National Institute for Health Research Health Protection Research Unit (NIHR HPRU) in Blood Borne and Sexually Transmitted Infections at University College London in partnership with Public Health England (PHE) and in collaboration with the London School of Hygiene and Tropical Medicine. The views expressed in this publication are those of the author(s) and not necessarily those of the NHS, the National Institute for Health Research, the Department of Health or Public Health England. Members of the Steering Committee of the NIHR HPRU: Caroline Sabin (Director), Anthony Nardone (PHE Lead), Catherine Mercer, Gwenda Hughes, Greta Rait, Jackie Cassell, William Rosenberg, Tim Rhodes, Kholoud Porter, and Samreen Ijaz.

## Potential conflicts of interest

JF has received grant support from Gilead Sciences and Merck. AG and KP have served on advisory boards for ViiV Healthcare. NN received honoraria for speaking and advising, travel grants and an institutional research grant from Bristol-Myers-Squibb, Janssen, ViiV Healthcare and Gilead Sciences. CO has received research grants, personal fees and non-financial support from Gilead Sciences, ViiV Healthcare, Bristol-Myers Squibb and MSD, and Janssen for advisory boards, lectureships and travel. All other authors declare that they have no conflict of interest.

## Author contributions

AG developed the discussion guide, scheduled and conducted the interviews, analysed the transcripts and wrote the manuscript. JF and KP conceived the study. JF recruited patients for interview, provided feedback on the discussion guide and early drafts of the manuscript. MG



For peer review only - http://bmjopen.bmj.com/site/about/guidelines.xhtml

provided social science advice regarding the analysis, interpretation and reporting of findings, double-coded a subset of the transcripts, and revised early drafts of the manuscript. SF, NN, AC, RG and CO organised and carried out recruitment of participants, and provided feedback on manuscript drafts. SC provided guidance on interpretation of preliminary findings and feedback on manuscript drafts. KP oversaw the development of fieldwork materials and early revisions of the manuscript. GH provided senior social science guidance on fieldwork, analysis, data interpretation and reporting, and revisions to early drafts of the manuscript. All authors contributed to and approved the current version of the manuscript.

## Data sharing statement

No additional data are available.

For peer review only



For peer review only - http://bmjopen.bmj.com/site/about/guidelines.xhtml

## References

1. Chau C, Kirwan P, Brown A, Gill N, Delpech V. HIV diagnoses, late diagnoses and numbers accessing treatment and care: 2016 report London: Public Health England; 2016 October 2016.
2. Skingsley A, Yin Z, Kirwan P, Croxford S, Chau C, Conti S, et al. HIV in the UK – Situation Report 2015: data to end 2014. London: Public Health England; 2015 November 2015.
3. Phillips AN, Cambiano V, Nakagawa F, Brown AE, Lampe F, Rodger A, et al. Increased HIV incidence in men who have sex with men despite high levels of ART-induced viral suppression: analysis of an extensively documented epidemic. PLoS One. 2013;8(2):e55312.
4. McCormack S, Dunn DT, Desai M, Dolling DI, Gafos M, Gilson R, et al. Pre-exposure prophylaxis to prevent the acquisition of HIV-1 infection (PROUD): effectiveness results from the pilot phase of a pragmatic open-label randomised trial. Lancet. 2016;387(10013):53-60.
5. Beyrer C, Baral SD, Collins C, Richardson ET, Sullivan PS, Sanchez J, et al. The global response to HIV in men who have sex with men. Lancet. 2016;388(10040):198-206.
6. Elam G, Macdonald N, Hickson FC, Imrie J, Power R, McGarrigle CA, et al. Risky sexual behaviour in context: qualitative results from an investigation into risk factors for seroconversion among gay men who test for HIV. Sex Transm Infect. 2008;84(6):473-7.
7. Macdonald N, Elam G, Hickson F, Imrie J, McGarrigle CA, Fenton KA, et al. Factors associated with HIV seroconversion in gay men in England at the start of the 21st century. Sex Transm Infect. 2008;84(1):8-13.
8. Desai M, Gill N, McCormack S, Dunn D, White E, Fox J, et al. Baseline predictors of HIV Infection in the no-PrEP group in the PROUD trial 22nd Annual Conference of the British HIV Association (BHIVA); 19-22 April 2016; Manchester2016.
9. Cohen MS, Chen YQ, McCauley M, Gamble T, Hosseinipour MC, Kumarasamy N, et al. Prevention of HIV-1 infection with early antiretroviral therapy. The New England journal of medicine. 2011;365(6):493-505.
10. Lampe F, Speakman A, Phillips A, Sherr L, Gilson R, Johnson M, et al. ART use, viral suppression, and sexual behaviour among HIV-diagnosed MSM in the UK: results from the Antiretrovirals, Sexual Transmission Risk and Attitudes (ASTRA) Study. Journal of the International AIDS Society. 2012;15((Suppl 4)):18143.
11. Gilbart VL, Simms I, Jenkins C, Furegato M, Gobin M, Oliver I, et al. Sex, drugs and smart phone applications: findings from semistructured interviews with men who have sex with men diagnosed with Shigella flexneri 3a in England and Wales. Sex Transm Infect. 2015;91(8):598-602.
12. Pufall E, Kall M, Shahmanesh M, Nardone A, Gilson R, Delpech V, et al. Chemsex and High-Risk Sexual Behaviours in HIV-Positive Men Who Have Sex With Men. Conference on Retroviruses and Opportunistic Infections; February 22–25 2016; Boston, Massachusetts2016.
13. Lorimer K, Flowers P, Davis M, Frankis J. Young men who have sex with men's use of social and sexual media and sex-risk associations: cross-sectional, online survey across four countries. Sex Transm Infect. 2016;92(5):371-6.
14. Dolling DI, Desai M, McOwan A, Gilson R, Clarke A, Fisher M, et al. An analysis of baseline data from the PROUD study: an open-label randomised trial of pre-exposure prophylaxis. Trials. 2016;17:163.
15. Baral S, Logie CH, Grosso A, Wirtz AL, Beyrer C. Modified social ecological model: a tool to guide the assessment of the risks and risk contexts of HIV epidemics. BMC Public Health. 2013;13:482.
16. Bronfenbrenner U. The ecology of human development: experiments by nature and design. . Cambridge, MA: Harvard University Press; 1979.
17. The UK register of HIV seroconverters: methods and analytical issues. UK register of HIV seroconverters (UKRHS) Steering Committee. Epidemiol Infect. 1996;117(2):305-12.



For peer review only - http://bmjopen.bmj.com/site/about/guidelines.xhtml

18. National Institute for Health Research. Health Protection Research Unit in Blood Borne and Sexually Transmitted Infections London: UCL Research Dept of Infection & Population Health; 2016 [Available from: http://bbsti.hpru.nihr.ac.uk/.
19. Ritchie J, Lewis J. Qualitative research practice: a guide for social science students and researchers. London: Sage; 2003.
20. Sandfort TG, de Graaf R, Ten Have M, Ransome Y, Schnabel P. Same-sex sexuality and psychiatric disorders in the second Netherlands Mental Health Survey and Incidence Study (NEMESIS-2). LGBT Health. 2014;1(4):292-301.
21. Wolitski J, Stall RD, Valdiserri RO. Unequal Opportunity. Health Disparities Affecting Gay and Bisexual Men in the United States. New York: Oxford University Press; 2008.
22. Koblin BA, Husnik MJ, Colfax G, Huang Y, Madison M, Mayer K, et al. Risk factors for HIV infection among men who have sex with men. AIDS. 2006;20(5):731-9.
23. Mimiaga MJ, O'Cleirigh C, Biello KB, Robertson AM, Safren SA, Coates TJ, et al. The effect of psychosocial syndemic production on 4-year HIV incidence and risk behavior in a large cohort of sexually active men who have sex with men. J Acquir Immune Defic Syndr. 2015;68(3):329-36.
24. Mustanski B, Garofalo R, Herrick A, Donenberg G. Psychosocial health problems increase risk for HIV among urban young men who have sex with men: preliminary evidence of a syndemic in need of attention. Ann Behav Med. 2007;34(1):37-45.
25. DiClemente RJ, Crittenden CP, Rose E, Sales JM, Wingood GM, Crosby RA, et al. Psychosocial predictors of HIV-associated sexual behaviors and the efficacy of prevention interventions in adolescents at-risk for HIV infection: what works and what doesn't work? Psychosom Med. 2008;70(5):598-605.
26. Singer M. AIDS and the health crisis of the U.S. urban poor; the perspective of critical medical anthropology. Soc Sci Med. 1994;39(7):931-48.
27. Bourne A, Reid D, Hickson F, Torres-Rueda S, Weatherburn P. Illicit drug use in sexual settings ('chemsex') and HIV/STI transmission risk behaviour among gay men in South London: findings from a qualitative study. Sex Transm Infect. 2015;91(8):564-8.
28. Elford J. HIV treatment optimism and high-risk sexual behaviour among gay men: the attributable population risk. AIDS. 2004;18(16):2216-7.
29. Kalichman SC, Eaton L, Cain D, Cherry C, Fuhrel A, Kaufman M, et al. Changes in HIV treatment beliefs and sexual risk behaviors among gay and bisexual men, 1997-2005. Health Psychol. 2007;26(5):650-6.
30. Cox J, Beauchemin J, Allard R. HIV status of sexual partners is more important than antiretroviral treatment related perceptions for risk taking by HIV positive MSM in Montreal, Canada. Sex Transm Infect. 2004;80(6):518-23.
31. Vanable PA, Ostrow DG, McKirnan DJ. Viral load and HIV treatment attitudes as correlates of sexual risk behavior among HIV-positive gay men. J Psychosom Res. 2003;54(3):263-9.
32. Ostrow DE, Fox KJ, Chmiel JS, Silvestre A, Visscher BR, Vanable PA, et al. Attitudes towards highly active antiretroviral therapy are associated with sexual risk taking among HIV-infected and uninfected homosexual men. AIDS. 2002;16(5):775-80.
33. Brennan DJ, Welles SL, Miner MH, Ross MW, Rosser BR, Positive Connections T. HIV treatment optimism and unsafe anal intercourse among HIV-positive men who have sex with men: findings from the positive connections study. AIDS Educ Prev. 2010;22(2):126-37.
34. Chelsea and Westminster Hospital NHS Foundation Trust. Dean Street PRIME London: 56 Dean Street, Chelsea and Westminster Hospital NHS Foundation Trust,; 2016 [Available from: http://prime.dean.st/.

For peer review only



For peer review only - http://bmjopen.bmj.com/site/about/guidelines.xhtml

# Tables

**Table 1. Demographic characteristics of 21 participants**

| Characteristic | Category | Number of participants |
|---|---|---|
| Age (years) | 20-29 | 6 |
| | 30-39 | 6 |
| | 40-49 | 5 |
| | ≥50 | 4 |
| Ethnicity | White | 19 |
| | BAME | 2 |
| Education | Secondary | 9 |
| | Higher education | 12 |
| Employment | Employed | 18 |
| | Unemployed | 3 |

BAME Black, Asian and Minority Ethnic

# Figure legends

**Figure 1. Adapted socio-ecological framework of factors influencing risk of HIV acquisition among recently infected MSM in London and Brighton**

<<insert figure 1 here>>

ART anti-retroviral therapy; PEP post-exposure prophylaxis
This adapted socio-ecological framework (15, 16) depicts individual- and interpersonal-level psycho-social risk factors within the wider community and structural environments. Factors within and between each layer interact to influence risk behaviours and risk of HIV acquisition.

**Figure 2. Examples of combinations of factors, applying a socio-ecological framework, which contributed to risk behaviours and/or HIV acquisition for three participants**

<<insert figure 2 here>>

ART anti-retroviral therapy; IPV intimate partner violence
Individual and interpersonal psycho-social factors are depicted in the inner circle, factors at the community-level are shown in the middle circle, and factors at the structural-level are depicted in the outer circle. In all three cases, factors from all levels contributed to HIV risk, although their relative importance varied. Case A and B both experienced a high density of individual-level psycho-social factors, which played a greater role than community or structural-level factors. These two cases also illustrate a contrasting balance of factors within the individual-level, with Case A having experienced psycho-social factors that accumulated from a young age, and Case B having experienced only more recent stressors. In case C, individual-level psycho-social factors contributed to a lesser extent than community and structural-level factors.



For peer review only - http://bmjopen.bmj.com/site/about/guidelines.xhtml

For peer review only

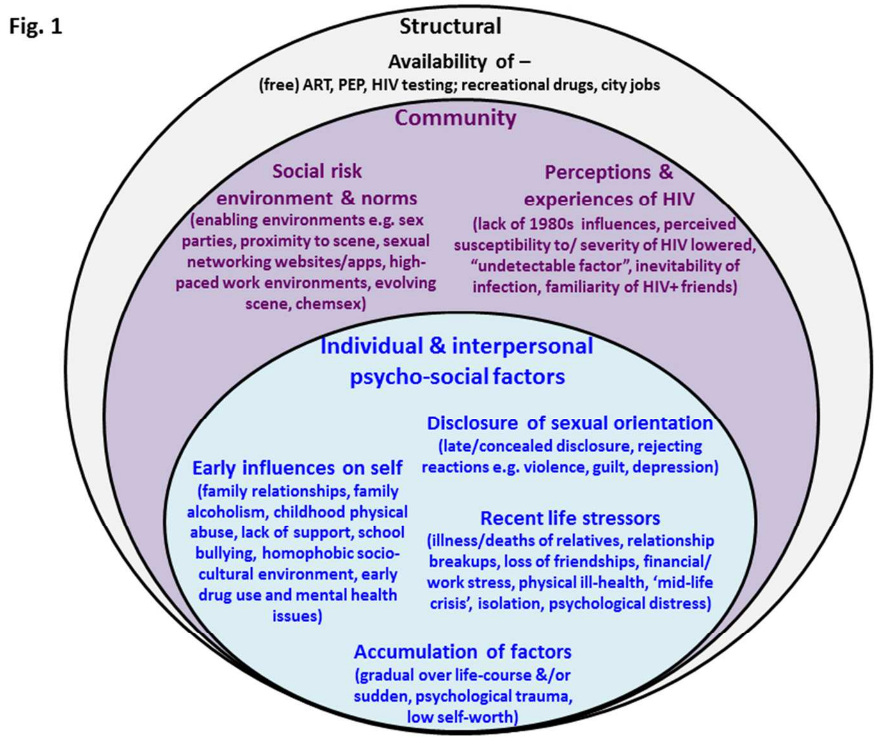


Figure 1. Adapted socio-ecological framework of factors influencing risk of HIV acquisition among recently infected MSM in London and Brighton

254x190mm (96 x 96 DPI)

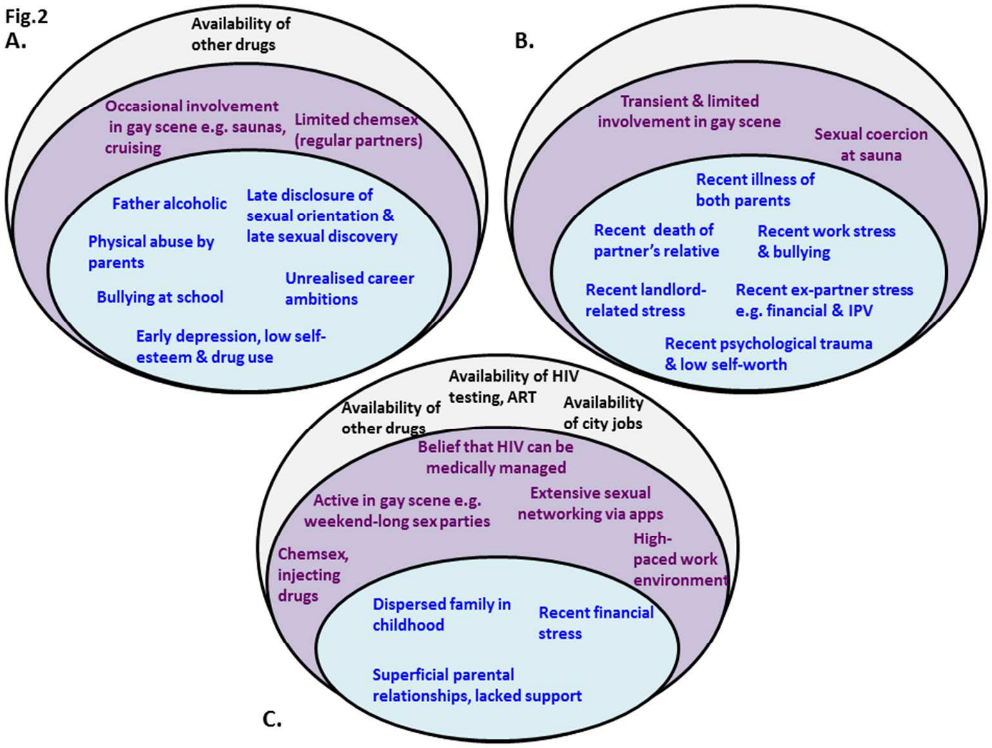


Figure 2. Examples of combinations of factors, applying a socio-ecological framework, which contributed to risk behaviours and/or HIV acquisition for three participants

254x190mm (96 x 96 DPI)

# Social factors related to HIV acquisition
# Interview discussion guide

## Introduction

- Thank for agreeing to participate
- Introduce self and organisation in collaboration with clinics
- Recap study objectives
- Remind participant they can stop the interview at any time or decline to answer
- Double check consent for recording the interview

## Ice-breaker & background

**Can you tell me a little bit about yourself and your background?**

- How old are you?
- Where are you from originally (born there)? A city or a rural area? Where do you live now? Who are you living with? How long have you been living there? If moved, when? From where? Why?
- Do you have family? Who? (Parents/ siblings – older or younger) Where are they living?
- What education have you received? University/higher education?
- Are you employed (or student)? What? Where? Since when?
- Are you in a relationship? Since when? Where is your partner from? If not in a relationship, previously in a relationship? When did this end? Relationships with men only, or men and women?
- Have you told anyone that you are gay or bisexual? When did you come out? How old were you then?

## Migration

**[If not originally from this city]**

**You said you moved to London/Brighton in yyyy. If you think back to that time, how were you feeling and how did you find this transition?**

- Moved alone or with someone else/ others?
- Sources of support?
- Any challenges?

For peer review only

## Circumstances around HIV infection

**Could you tell me about your life in the last year or so, just before you discovered you were HIV positive?**

- Monogamous/exclusive sexual relationship(s)?
- Or more casual sexual partners/ dating? Nature and number of partners? Preferred casual partners, or seeking long-term relationship?
- Where and how you met? Social activities? Clubs, bars, sex clubs, saunas, cruising (public environment)? Any change in habits?
- Recent travel (sex) abroad? (e.g. Amsterdam, Paris, Berlin)
- Internet, dating apps, other social media? Any change in use?
- Experienced sexual violence (in last year, or before, e.g. forced you to have sex)? Or other physical abuse (e.g. he hit you)? Shouting or threatening you (emotional abuse)? Any other frightening behaviour?
- Friends?
- How were you feeling emotionally (psychological well-being)? Self-confidence? What triggered any change?
- Health?
- Work?
- Financial situation?
- Home and family?
- Moving/re-location?
- Lifestyle?
- Drug or alcohol use? If yes, how did you get involved in/ what triggered this? Why?
- Any other challenges faced during this time?
- Negative reactions towards your sexuality/being gay? (Stigma (homophobia))

**Do you know when you might have caught HIV? How? From whom?**

- HIV-status and nature of sexual partner(s)?
- Sexual behaviour (condom use, breakage, receptive/insertive partner, ejaculation?)
- Were you (ever) forced into sex?
- To what extent did you see this [e.g. unprotected sex] as a risk to your sexual health? Why? (Risk perception and attitudes to unprotected sex)
- Did you try to manage this risk [e.g. sex without condom]? How? Any challenges? Ever discussed this with anyone – partners, friends, health or other support services? (Risk management)
- How were you feeling at this time?
- Drug (e.g. poppers, ecstasy) or alcohol use before or during sex?
- How did you (or other HIV-positive peers) become involved in [drug use/ high risk practices/ gay social scene]?
- What (other) factors do you think contributed (to risk behaviour and getting infected)? (Probe for social factors above) How?
- Why do you think you got HIV *now*? What factors or events triggered this?

For peer review only

## Overcoming challenges and staying HIV-negative

**You mentioned […] were difficulties you faced at this time.  How might any of these difficulties/events have been overcome?**

- Support services?  What exactly?  By whom?  How would this have helped?

**What would have helped you to stay HIV negative?**

## Previous STI/HIV testing patterns and counselling

**Can you tell me about any contact you had with health services for STI testing *before* you were diagnosed with HIV?**

- How many times did you get tested for HIV?
- How did you react to these (negative) results? Effect on sexual behaviour?
- Diagnosed with other STIs?  Which? When?
- Experiences with using these services?
- Perceptions of the counselling given (including drug use counselling). What advice given? Helpful or not? How easy to understand the advice or put it into practice? Timing of advice (started receiving in time/ too late?)
- Any other advice or support (clinic-based or other, e.g. social groups, internet-based) you received, or wish you had received?

## Initial perceptions of HIV care and support services

**Some people can find it difficult to continue attending clinics or to take treatment in the long-term.  Based on your initial experiences of the HIV services you have received and your personal life at the moment, do you anticipate any difficulties with being able to continue attending?**

- What difficulties?
- Disclosure (planned/already? How did that go?) Experienced/anticipated negative reactions (stigma)?
- Probe for any of the social or psychological factors discussed above
- Attended/ experiences or perceptions of other non-clinic support services?
- What would help you to continue attending/ follow clinical advice?

## END OF INTERVIEW

- Thank participant for their time
- Reassure confidentiality of the discussion
- Provide information on relevant support services

For peer review only

Table 1

**Standards for Reporting Qualitative Research (SRQR)[a]**

| No. | Topic | | Item |
|---|---|---|---|
| | **Title and abstract** | | |
| S1 | Title | page 1 | Concise description of the nature and topic of the study Identifying the study as qualitative or indicating the approach (e.g., ethnography, grounded theory) or data collection methods (e.g., interview, focus group) is recommended ✓ |
| S2 | Abstract | page 2 | Summary of key elements of the study using the abstract format of the intended publication; typically includes background, purpose, methods, results, and conclusions ✓ |
| | **Introduction** | page 4-5 | |
| S3 | Problem formulation | | Description and significance of the problem/phenomenon studied; review of relevant theory and empirical work; problem statement ✓ |
| S4 | Purpose or research question | | Purpose of the study and specific objectives or questions ✓ |
| | **Methods** | page 6-8 | |
| S5 | Qualitative approach and research paradigm | p 7 | Qualitative approach (e.g., ethnography, grounded theory, case study, phenomenology, narrative research) and guiding theory if appropriate; identifying the research paradigm (e.g., postpositivist, constructivist/ interpretivist) is also recommended; rationale[b] ✓ |
| S6 | Researcher characteristics and reflexivity | p 7 p 26 | Researchers' characteristics that may influence the research, including personal attributes, qualifications/experience, relationship with participants, assumptions, and/or presuppositions; potential or actual interaction between researchers' characteristics and the research questions, approach, methods, results, and/or transferability ✓ |
| S7 | Context | p 6 | Setting/site and salient contextual factors; rationale[b] ✓ |
| S8 | Sampling strategy | p 6 | How and why research participants, documents, or events were selected; criteria for deciding when no further sampling was necessary (e.g., sampling saturation); rationale[b] ✓ |
| S9 | Ethical issues pertaining to human subjects | p 8 | Documentation of approval by an appropriate ethics review board and participant consent, or explanation for lack thereof; other confidentiality and data security issues ✓ |
| S10 | Data collection methods | p 7 | Types of data collected; details of data collection procedures including (as appropriate) start and stop dates of data collection and analysis, iterative process, triangulation of sources/methods, and modification of procedures in response to evolving study findings; rationale[b] ✓ |
| S11 | Data collection instruments and technologies | p 7 | Description of instruments (e.g., interview guides, questionnaires) and devices (e.g., audio recorders) used for data collection; if/how the instrument(s) changed over the course of the study ✓ |
| S12 | Units of study | p6-7 p 9 | Number and relevant characteristics of participants, documents, or events included in the study; level of participation (could be reported in results) ✓ |
| S13 | Data processing | p7-8 | Methods for processing data prior to and during analysis, including transcription, data entry, data management and security, verification of data integrity, data coding, and anonymization/deidentification of excerpts ✓ |
| S14 | Data analysis | p 7-8 | Process by which inferences, themes, etc., were identified and developed, including the researchers involved in data analysis; usually references a specific paradigm or approach; rationale[b] ✓ |
| S15 | Techniques to enhance trustworthiness | p 7-8 | Techniques to enhance trustworthiness and credibility of data analysis (e.g., member checking, audit trail, triangulation); rationale[b] ✓ |
| | **Results/findings** | | |
| S16 | Synthesis and interpretation | p 9-23 | Main findings (e.g., interpretations, inferences, and themes); might include development of a theory or model, or integration with prior research or theory ✓ |
| S17 | Links to empirical data | p 9-23 | Evidence (e.g., quotes, field notes, text excerpts, photographs) to substantiate analytic findings ✓ |
| | **Discussion** | | |
| S18 | Integration with prior work, implications, transferability, and contribution(s) to the field | p 24-27 | Short summary of main findings; explanation of how findings and conclusions connect to, support, elaborate on, or challenge conclusions of earlier scholarship; discussion of scope of application/ generalizability; identification of unique contribution(s) to scholarship in a discipline or field ✓ |
| S19 | Limitations | p 26 | Trustworthiness and limitations of findings ✓ |

(*Table continues*)



168x233mm (300 x 300 DPI)

Table 1
(Continued)

| No. | Topic | | Item | |
|---|---|---|---|---|
| | **Other** | | | |
| S20 | Conflicts of interest | p28 | Potential sources of influence or perceived influence on study conduct and conclusions; how these were managed | ✓ |
| S21 | Funding | p 28 | Sources of funding and other support; role of funders in data collection, interpretation, and reporting | ✓ |

[a]The authors created the SRQR by searching the literature to identify guidelines, reporting standards, and critical appraisal criteria for qualitative research; reviewing the reference lists of retrieved sources; and contacting experts to gain feedback. The SRQR aims to improve the transparency of all aspects of qualitative research by providing clear standards for reporting qualitative research.

[b]The rationale should briefly discuss the justification for choosing that theory, approach, method, or technique rather than other options available, the assumptions and limitations implicit in those choices, and how those choices influence study conclusions and transferability. As appropriate, the rationale for several items might be discussed together.

should provide evidence (e.g., examples, quotes, or text excerpts) to substantiate the main analytic findings.[20,29]

**Discussion.** The discussion of qualitative results will generally include connections to existing literature and/or theoretical or conceptual frameworks, the scope and boundaries of the results (transferability), and study limitations.[10–12,28] In some qualitative traditions, the results and discussion may not have distinct boundaries; we recommend that authors include the substance of each item regardless of the section in which it appears.

## Discussion

The purpose of the SRQR is to improve the quality of reporting of qualitative research studies. We hope that these 21 recommended reporting standards will assist authors during manuscript preparation, editors and reviewers in evaluating a manuscript for potential publication, and readers when critically appraising, applying, and synthesizing study findings. As with other reporting guidelines,[35–37] we anticipate that the SRQR will evolve as it is applied and evaluated in practice. We welcome suggestions for refinement.

Qualitative studies explore "how?" and "why?" questions related to social or human problems or phenomena.[10,38] Purposes of qualitative studies include understanding meaning from participants' perspectives (How do they interpret or make sense of an event, situation, or action?); understanding the nature and influence of the context surrounding events or actions; generating theories about new or poorly understood events, situations, or actions; and understanding the processes that led to a desired (or undesired) outcome.[38] Many different approaches (e.g., ethnography, phenomenology, discourse analysis, case study, grounded theory) and methodologies (e.g., interviews, focus groups, observation, analysis of documents) may be used in qualitative research, each with its own assumptions and traditions.[1,2] A strength of many qualitative approaches and methodologies is the opportunity for flexibility and adaptability throughout the data collection and analysis process. We endeavored to maintain that flexibility by intentionally defining items to avoid favoring one approach or method over others. As such, we trust that the SRQR will support all approaches and methods of qualitative research by making reports more explicit and transparent, while still allowing investigators the flexibility to use the study design and reporting format most appropriate to their study. It may be helpful, in the future, to develop approach-specific extensions of the SRQR, as has been done for guidelines in quantitative research (e.g., the CONSORT extensions).[37]

### Limitations, strengths, and boundaries

We deliberately avoided recommendations that define methodological rigor, and therefore it would be inappropriate to use the SRQR to judge the quality of research methods and findings. Many of the original sources from which we derived the SRQR were intended as criteria for methodological rigor or critical appraisal rather than reporting; for these, we inferred the information that would be needed to evaluate the criterion. Occasionally, we found conflicting recommendations in the literature (e.g., recommending specific techniques such as multiple coders or member checking to demonstrate trustworthiness); we resolved these conflicting recommendations through selection of the most frequent recommendations and by consensus among ourselves.

Some qualitative researchers have described the limitations of checklists as a means to improve methodological rigor.[13] We nonetheless believe that a checklist for reporting standards will help to enhance the transparency of qualitative research studies and thereby advance the field.[29,39]

Strengths of this work include the grounding in previously published criteria, the diversity of experience and perspectives among us, and critical review by experts in three countries.

### Implications and application

Similar to other reporting guidelines,[35–37] the SRQR may be viewed as a starting point for defining reporting standards in qualitative research. Although our personal experience lies in health professions education, the SRQR is based on sources originating in diverse health care and non-health-care fields. We intentionally crafted the SRQR to include various paradigms, approaches, and methodologies used in qualitative research. The elaborations offered in



164x233mm (300 x 300 DPI)

BMJ Open

# A qualitative study exploring the social and environmental context of recently-acquired HIV infection among men who have sex with men in South-East England

| | |
|---|---|
| Journal: | *BMJ Open* |
| Manuscript ID | bmjopen-2017-016494.R1 |
| Article Type: | Research |
| Date Submitted by the Author: | 23-Jun-2017 |
| Complete List of Authors: | Gourlay, Annabelle; University College London, Department of Infection and Population Health<br>Fox, Julie; King's College London, Guy's and St Thomas' NHS Trust<br>Gafos, Mitzy ; University College London, MRC Clinical Trials Unit; London School of Hygiene and Tropical Medicine<br>Fidler, Sarah; Imperial College London, Department of Medicine<br>Nwokolo, Nneka; Chelsea and Westminster Hospital NHS Foundation Trust, 56 Dean Street, Department of HIV/GUM<br>Clarke, Amanda; Brighton and Sussex University Hospital , Elton John Centre<br>Gilson, Richard; University College London, Department of Infection and Population Health<br>Orkin, Chloe; Barts Health NHS Trust, Ambrose King Centre, Royal London Hospital<br>Collins, Simon; HIV i-Base<br>Porter, Kholoud; University College London, Department of Infection and Population Health<br>Hart, Graham; University College London, Department of Infection & Population Health |
| <b>Primary Subject Heading</b>: | HIV/AIDS |
| Secondary Subject Heading: | Qualitative research, Sexual health, Mental health, Epidemiology |
| Keywords: | HIV & AIDS < INFECTIOUS DISEASES, Epidemiology < INFECTIOUS DISEASES, Public health < INFECTIOUS DISEASES, MENTAL HEALTH, QUALITATIVE RESEARCH |
| | |

SCHOLARONE™
Manuscripts

# A qualitative study exploring the social and environmental context of recently-acquired HIV infection among men who have sex with men in South-East England

Annabelle Gourlay[1,2], Julie Fox[3], Mitzy Gafos[1], Sarah Fidler[4], Nneka Nwokolo[5], Amanda Clarke[6], Richard Gilson[1], Chloe Orkin[7], Simon Collins[8], Kholoud Porter[1,2] and Graham Hart[1]

**Affiliations**:
1 Faculty of Population Health Sciences, University College London, London, UK
2 National Institute for Health Research Health Protection Research Unit in Blood Borne and Sexually Transmitted Infections, UK
3 Guys and St Thomas' NHS Trust/ King's College London, London, UK
4 Imperial College London, London, UK
5 56 Dean Street, Department of HIV/GUM, Chelsea and Westminster Hospital NHS Foundation Trust, London, UK
6 Elton John Centre, Royal Sussex County Hospital, Brighton and Sussex University Hospital, Brighton, UK
7 Ambrose King Centre, Royal London Hospital, Barts Health NHS Trust, London, UK
8 HIV i-Base, London, UK

**Correspondence to**: Dr Annabelle Gourlay, Department of Infection and Population Health, Faculty of Population Health Sciences, University College London, Mortimer Market Centre, off Capper Street, London WC1E 6JB. Telephone: +44 (0)20 7679 7611. Email: a.gourlay@ucl.ac.uk

Abstract word count (283) Manuscript word count (5982) Tables (1) Figures (2)

**Suggested running head**: Social context of recent HIV infections among MSM in South-East England

For peer review only

## Abstract

**Objectives**: A key UK public health priority is to reduce HIV incidence among gay and other men who have sex with men (MSM). This study aimed to explore the social and environmental context in which new HIV infections occurred among MSM in London and Brighton in 2015.

**Design**: A qualitative descriptive study, comprising in-depth interviews, was carried out as a sub-study to the UK Register of HIV Seroconverters cohort: an observational cohort of individuals whose date of HIV seroconversion was well-estimated. An inductive thematic analysis was conducted in NVivo, guided by a socio-ecological framework.

**Setting**: Participants were recruited from 6 HIV clinics in London and Brighton. Fieldwork was conducted between January and April 2015.

**Participants**: All MSM eligible for the UK Register Seroconverter cohort (an HIV-positive antibody test result within 12 months of their last documented HIV-negative test, or other laboratory evidence of HIV seroconversion) diagnosed within the past 12 months and aged ≥18 were eligible for the qualitative sub-study. 21 MSM participated, aged 22 – 61 years and predominantly white.

**Results**: A complex interplay of factors, operating at different levels, influenced risk behaviours and HIV acquisition. Participants saw risk as multifactorial but the relative importance of factors varied for each person. Individual psycho-social factors, including personal history, recent life stressors and mental health, enhanced vulnerability towards higher risk situations, while features of the social environment, such as chemsex and social media, and prevalent community beliefs regarding treatment and HIV normalisation, encouraged risk-taking.

**Conclusions**: Recently-acquired HIV infection among MSM reflects a complex web of factors operating at different levels. These findings point to the need for multi-level interventions to reduce the risk of HIV acquisition among high risk MSM in the UK and similar settings.



For peer review only - http://bmjopen.bmj.com/site/about/guidelines.xhtml

**Strengths and limitations of this study**

- This study provides important insights into the social and environmental contexts for recent HIV infections among gay and other men who have sex with men (MSM) in the United Kingdom, where reducing HIV incidence remains a public health priority.
- A unique feature was the recruitment of participants known to have recently acquired HIV infection, maximising their ability to recall contextual information.
- The primary limitation is the generalisability of findings that may not extend to MSM in other areas or ethnic groups, given participants were exclusively recruited from London/ Brighton and most were white. Nonetheless, our findings are likely to apply to similar UK or international cities with large populations of MSM.
- Social desirability bias is a further possible limitation, despite many respondents disclosing sensitive personal issues.

**Keywords**

HIV; United Kingdom; MSM; recent HIV infection; sexual behaviour; mental health; chemsex

For peer review only



For peer review only - http://bmjopen.bmj.com/site/about/guidelines.xhtml

## Background

Gay men and other men who have sex with men (MSM) accounted for more than half of individuals newly-diagnosed with HIV in the UK in 2015 (1), with evidence of a rising trend in HIV incidence in recent years and continued high rates of transmission (2-4). Understanding the context in which new infections occur in this key population therefore remains a priority (5).

Qualitative and quantitative research conducted in the early 2000s identified behavioural factors associated with the risk of HIV acquisition among MSM in the UK, including condomless sex and an increasing number of partners, as well as the use of poppers (nitrite inhalants) and psychological factors such as depression (6, 7). However, psycho-social factors were not explored or reported in detail and their importance over a decade later remains unclear. More recent analyses using data from the PROUD trial identified ≥2 condomless sex partners in the last 90 days and having a bacterial rectal sexually transmitted infection (STI) in the previous year as the strongest risk factors for HIV acquisition (8). However, qualitative insights into behavioural or social factors associated with HIV acquisition were not available.

Important changes have occurred over the last decade in HIV prevention and the social environment in which MSM interact. New medical interventions, such as the use of antiretroviral treatment (ART) as prevention (9), pre-exposure prophylaxis (PrEP) (4) and the availability of HIV self-testing kits are likely to influence perceptions of risk, risk behaviours and risk of HIV acquisition. For example, sexual behaviours might be influenced by the growing awareness that having an undetectable viral load (VL) dramatically reduces the risk of HIV transmission (10). The context of sex between men has also changed dramatically, with the widespread availability of sexual networking through websites or mobile phone-based applications ('apps') and greater availability of psychoactive drugs used for 'chemsex' (specifically mephedrone, γ-hydroxybutrate/γ-butyrolactone (GHB/GBL) or crystal



For peer review only - http://bmjopen.bmj.com/site/about/guidelines.xhtml

methamphetamine, taken intentionally to enhance sex). Evidence is emerging of high levels of engagement in these behaviours (11-13) with almost one in three HIV-positive MSM participating in a UK-based HIV clinic survey and 44% of HIV-negative MSM enrolled in the PROUD PrEP study reporting chemsex in the previous year (12, 14). However, the specific role of chemsex or social media and sexual networking in the acquisition of HIV or other STIs is less clear.

Relatively little is known about the social context of and behavioural responses to medical interventions, chemsex drugs or sexual networking media. Research is also lacking on how these factors, importantly, interrelate or interact with individual-level psycho-social factors, such as mental health, to influence HIV risk. Baral et al argued that, although epidemiological studies have traditionally focussed on individual-level risk factors, it is essential to capture data that characterise multiple levels of HIV risk, including higher order social and structural-level risks (15). Levels of HIV risk and complex associations of factors at different levels can be appropriately investigated and depicted using socio-ecological frameworks, in which individuals and individual-level risks are conceptualised as part of the wider community and policy environment (Figure 1) (15, 16).

Through interviews with MSM who had recently acquired HIV infection, we set out to: i) explore the social and environmental contexts in which HIV acquisition occurred among MSM in London and Brighton in 2015; ii) investigate how these contexts influenced HIV risk; and iii) inform the design of public health interventions to help MSM reduce the risk of HIV acquisition.

For peer review only



For peer review only - http://bmjopen.bmj.com/site/about/guidelines.xhtml

## Methods

***The UK Register cohort study***

We conducted a qualitative sub-study to the UK Register of HIV Seroconverters: an observational cohort of individuals whose date of HIV seroconversion was well-estimated (17). Eligible individuals were ≥16 years of age and had an HIV-positive antibody test result within 12 months of their last documented HIV-negative test, or other laboratory evidence of HIV seroconversion. Dates of seroconversion were estimated based on the latter, or the mid-point between last HIV-negative and first HIV-positive test result.

***Qualitative study design***

The qualitative study was undertaken within the National Institute for Health Research Health Protection Research Unit (NIHR HPRU) in Blood Borne and Sexually Transmitted Infections (http://bbsti.hpru.nihr.ac.uk/) (18). We used a qualitative descriptive design (19) guided by socio-ecological theory (15, 16), employing in-depth interview data collection techniques and a thematic content analysis.

***Selection and recruitment of participants for interview***

All MSM eligible for the UK Register, diagnosed within the past 12 months, aged ≥18 years, and recruited from 6 centres in London and Brighton, South-East England, were eligible for the qualitative sub-study. Centres were selected based on levels of recruitment to the cohort study and location, aiming to include areas with differing patient demographics. We restricted recruitment to these areas for logistical reasons and in view of their importance in the UK HIV epidemic, with over half of new HIV diagnoses among MSM in the UK made in London (2) and large MSM populations in both cities. HIV doctors undertook recruitment, explaining the study aims and providing information sheets. Only individuals who were willing and felt able to undertake the interview were recruited.



For peer review only - http://bmjopen.bmj.com/site/about/guidelines.xhtml

Participants who gave their written consent to participate were contacted by an independent researcher (AG), or by a research nurse, as preferred, to schedule the interview.

Recruitment occurred between January-March 2015 until a minimum of 20 participants had been recruited and data saturation was reached on most themes. Thirty-six men were invited, five declined and six expressed interest but did not return to give consent. Of 25 who consented, one could not be contacted and three were unavailable for interview in the time-frame. Two men were subsequently excluded from the cohort as their last negative test result could not be verified, but were included in the qualitative study.

***Data collection***

In-depth interviews were conducted between February and April 2015 by an independent researcher (AG). Interviews took place in private rooms at each clinic and lasted 1-1.5 hours. Participants were advised they could decline to answer questions or stop the interview at any time.

The interviews followed a discussion guide (Supplement 1) including personal background, moving to London/Brighton and experiences of this transition, if applicable, life in recent years before HIV diagnosis, relationships, and perceptions of the circumstances at the time of HIV infection. The guide was adapted during fieldwork to accommodate emerging themes.

Interviews were audio-recorded using a password-protected digital recorder and summary notes were written after each interview. Recordings were transcribed verbatim and audio-files destroyed thereafter. Transcripts were password-protected and stored on a secure computer network with restricted permissions. Transcripts were labelled with codes (e.g. 'participant_A_date') and data reported anonymously.



For peer review only - http://bmjopen.bmj.com/site/about/guidelines.xhtml

***Data analysis***

Summary notes informed preliminary analyses. A thematic analysis was then conducted on the interview transcripts using NVivo 11 software. Data were indexed and categorised to construct an analytic thematic framework. An inductive approach was used, further aided by topics on the discussion guide. A sub-sample (>10%) of transcripts was verified by a second researcher (MG) by constructing thematic maps and comparing themes, which showed a high level of consistency. Any discrepancies were resolved through discussions, resulting in some new categories being identified and added at this stage and some categories being refined. The revised framework was then re-applied to all transcripts. Data were subsequently summarised using thematic matrices (20) in Microsoft Excel to enable a comparison of themes within and across cases (interviews). The research team discussed themes at several stages of the fieldwork and analysis. A socio-ecological model was used to organise themes and data presentation (Figure 1) (15, 16). Interview quotations that best described the themes were selected for presentation, whilst also aiming to incorporate examples from most participants and all age groups.

***Ethics approvals***

The qualitative study was approved by West Midlands, South Birmingham National Research Ethics Service, as an amendment to the UK Register protocol (reference 04/Q207155). Participants gave informed signed consent, including for the audio-recording of interviews.

For peer review only



For peer review only - http://bmjopen.bmj.com/site/about/guidelines.xhtml

## Results

Interviews were completed with 21 participants, within 5 months, on average, of their HIV diagnosis and 6 months from their estimated date of HIV seroconversion. Participants were aged between 22 and 61 with a median age of 38, were mostly white, well educated, and employed (Table 1).

We first report the psycho-social factors, described by the majority of participants, at the individual and interpersonal-level that increased vulnerability to risk, including HIV acquisition. We then present themes at the community and structural-level, including the social risk environment and community perceptions of HIV, which enabled risk-taking. The relative importance of factors at each level and the way they came together to influence risk are illustrated in the final section and in Figure 1.

### *Individual and interpersonal*

Analysis of the in-depth interviews identified four main themes at the individual and interpersonal level. 'Early influences on self' encompasses childhood experiences associated with family - often characterised by poor relationships - school, and participants' broader socio-cultural context growing up. 'Disclosure of sexual orientation' comprises the frequent rejecting reactions participants faced when disclosing their sexual orientation to others, and the inner struggles and repercussions associated with initial concealment of sexual identity. 'Recent life stressors' are defined as distressing and sometimes severe events, such as deaths of relatives, experienced by many participants in the few years before their HIV diagnosis. The experience of multiple psycho-social issues, either as a series of sudden recent stressors, or gradually since childhood, is captured in the fourth theme termed the 'accumulation of psycho-social risk factors'. We describe each theme in turn, drawing out their impacts on psychological wellbeing, then demonstrate how these



For peer review only - http://bmjopen.bmj.com/site/about/guidelines.xhtml

For peer review only

intermediary psychological outcomes affected risk behaviours, including risk of HIV infection, in the final sub-section.

***Early influences on self***

Experiences during childhood, within the family, school and the broader socio-cultural environment, had early and long-lasting impacts on mental health, drug use, and support structures. While some respondents reported strong relationships with family members, the majority described dysfunctional or superficial relationships with their parent(s), characterised by a lack of love or attention, abandonment, arguments, physical or verbal abuse, or parents/ siblings with alcohol or mental health issues. Many participants described how this led to low self-esteem, lack of confidence, insecurity, unhappiness, anxiety, depression or self-destructive behaviours.

> *My father was...an alcoholic and he used to beat my mother and me...That may have had some impact on how destructive one is, and the fact I never had any unconditional love is something that I have struggled with in adulthood.* (Aged 40-49)

Family alcoholism occasionally influenced participants' own drug or alcohol habits, while early separation from family contributed to loneliness and early drug use for some participants. School-based bullying was recalled by several participants, with similar psychological repercussions.

> *I: And were you able to identify any reasons behind that [anxiety]?*
>
> *R: Yeah, lots and lots: mum, dad, school...I was far too shy a kid for boarding school. I'd get squished in the corner.* (Aged 20-29)



For peer review only - http://bmjopen.bmj.com/site/about/guidelines.xhtml

A few men were raised in cultures where gay men were commonly stigmatised or discriminated against, including highly religious contexts, abroad and/or in rural settings, which shaped self-perceptions regarding sexual identity and resulted in repressed sexuality or low self-esteem.

> *It is quite difficult to understand in this day and age how prejudiced and narrow minded society was…where if I had been caught being gay…the school would have expelled me…never mind social views…So I was completely closeted and it became a huge frustration. (Aged 40-49)*

***Disclosure of sexual orientation***

Most participants had disclosed their sexual orientation as teenagers or young adults. A few had initially resisted or concealed their sexual identity and discussed the psychological implications of self-denial.

> *I was trying to change myself into a straight man...It didn't quite work out...I was basically on my own. My family didn't want to see me again…So the signs of depression were already there.* (Aged 40-49)

Several self-identified and came out as gay in their mid-twenties or thirties. For some, early concealment and/or late disclosure were associated with resentment regarding missed opportunities, prompting a desire for sexual exploration.

> *Growing up in an environment where you are getting to know yourself quite late, you get to...thinking about experiences and seeing other sexual stuff that you might not have needed to think about before because you are a bit behind...You know, am I missing out on stuff?* (Aged 40-49)



For peer review only - http://bmjopen.bmj.com/site/about/guidelines.xhtml

Participants had commonly experienced negative reactions to their disclosure. Several described 'traumatic' responses, including physical violence and rejection, which led to feelings of guilt or depression, frayed family relationships and weakened support networks.

> *It [coming out] was quite traumatic at the time. My mum has never knowingly under-reacted to anything.* (Aged 40-49)

***Recent life stressors***

Recent stressful events experienced prior to HIV diagnosis caused psychological distress for many participants. These encompassed severe illnesses or deaths of relatives, relationship break-ups, partner/ sexual violence, loss of friendships, and bouts of physical ill-health.

> *I: Can you identify a particular trigger for that [depression]?*
>
> *R: I think it was a consequence of events...It was...falling in love with someone who I couldn't be with...splitting up with my long term partner.* (Aged 40-49)

A number reported financial or work stress, including harassment, as key life stressors. Alcohol or drug use, as well as loneliness and isolation were common outcomes, associated with fragmentation of support networks.

> *About 2013, that's when everything started to get sour at work...a lot of stress, being really unhappy...relying on drinking.* (Aged 30-39)

A few middle-aged men described a 'forty-something crisis', unfulfilled ambitions, or wavering self-worth.



For peer review only - http://bmjopen.bmj.com/site/about/guidelines.xhtml

For peer review only

*I mean it probably was the perfect storm you know, they [drugs] got me at a time…mid-forties when I wasn't that secure, there were a few issues, I was looking for fun...it was an escape and it seemed at the time that it was...enjoyable.* (Aged 50-59)

***Accumulation of psycho-social risk factors***

Several individuals were exposed to multiple psycho-social risk factors from a young age which accumulated gradually.

*We have to go back to my childhood...I think everything [experiences, e.g. childhood physical abuse, early concealment of sexual identity, depression, mother's recent death] adds a weight and it's been add and add and add to a point where I lost control...I decided to let myself go.* (Aged 40-49)

In other cases, or additionally, a series of de-stabilising, traumatic events occurred suddenly.

*The year before last it was like every other week something was happening or somebody was dying and my mum had a stroke, my dad had a heart attack, [partner's] dad was involved in a really bad car crash and nearly died and...then his auntie was diagnosed with motor neurone and it was like "anything else?"...It was just so much.* (Aged 30-39)

In both scenarios, individuals became overwhelmed, unable to cope, lost control or experienced psychological trauma, including low self-worth, loss of purpose, depression and suicidal thoughts.



For peer review only - http://bmjopen.bmj.com/site/about/guidelines.xhtml

> *I was probably overwhelmed, you know. I wasn't in a stable place, I wasn't in a stable relationship, I wasn't stable financially and I had just suffered some pretty serious losses in terms of my immediate family. It was kind of all over the place really.* (Aged 40-49)

***Impacts on risk behaviour***

Psychological impacts sometimes directly affected sexual behaviour, for example, sex sought for escapism or self-validation. One man, who said he never "*felt nurtured*" by his parents explained:

> *I always need validation from people...and that manifests itself in a sexual context.* (Aged 40-49)

Loneliness and a desire for intimacy also prompted condomless sex. For example, one man who described himself as previously "*very strict with sex practices*" said:

> *It was probably because of the breakup of my relationship I was just feeling a need to be close to people, and that often came out in me choosing to have sex, unsafe without a condom.* (Aged 30-39)

Some individuals deliberately put themselves at risk, for example having condomless sex, including with HIV-positive partners, to self-harm or re-gain control, but did not indicate this was a recent change in their behaviour.

> *A lot of the difficulties I have...were about feeling controlled or not in control…so I knew what the risks were...but it was my choice, my decision [to have condomless sex with known HIV-positive partners].* (Aged 30-39)



For peer review only - http://bmjopen.bmj.com/site/about/guidelines.xhtml

Changes in attitudes to risk were also brought about by emotional trauma. A few participants described re-evaluating the potential costs of unsafe sex and the risk of HIV-infection, relative to other more important life events, or justifying their risk-taking during transient periods of psychological distress. Others cared so little for themselves that getting HIV seemed inconsequential.

> *I didn't value my life...Because so much had happened and I'd been through so much in the past 3, 4, 5 years with...break ups and losing everything and emotional things and deaths and God knows what else, it almost becomes a bit 'all my life has just been so crap anyway what's the point, do I really care if I get it [HIV] anyway?'.* (Aged 30-39)

For peer review only

### *Community and structural*

At the community and structural levels, two key themes emerged. 'Social risk environment and norms' captures the temptations of the evolving social scene, for example sexual networking via social media and normalisation of risk-taking behaviours such as chemsex, interlaced with structural factors such as availability of recreational drugs, that exposed individuals to potential risks including HIV acquisition. The second theme, 'community perceptions and experiences of HIV', describes shifting community attitudes towards HIV, influenced by structural factors such as the availability of ART, PEP and HIV testing, and the impacts in terms of reduced risk perceptions and willingness to engage in risk behaviours.

#### *Social risk environment and norms*

Participants were attracted to London and Brighton for the open-minded culture, freedom, and social opportunities offered, although most had lived in these urban areas for a number of years. Almost all had socialised or met sex partners at saunas, clubs, private sex parties, 'chill-outs' (after-



For peer review only - http://bmjopen.bmj.com/site/about/guidelines.xhtml

parties, typically involving drugs and/or group sex), or cruising grounds. For a few city workers, the intensity of partying was stimulated by challenging and high-paced work environments. Temptations of the scene were hard to resist for some men, while physical proximity to venues increased social opportunities and often exacerbated high-risk behaviours. Many participants talked about the excesses of partying on the scene and roughly half talked about their experiences of weekend-long binges of drugs and sex.

> *You go to Vauxhall [district of South London with a high-density of gay venues] on a Friday night as a gay man and don't come home until five days later. I think there is so much to tempt young people these days, I include myself in that.* (Aged 20-29)

Social media, including geo-locational sexual networking apps, also provided convenient access to multiple sexual partners for many participants, regardless of age. Social media were often used deliberately in pursuit of sex and/or drugs and widened sexual networks including with HIV-positive men.

> *The introduction of gay apps just makes the sex so much more promiscuous...I think people get a rush from it. I probably did at the time. These apps...just make it so much more convenient. They had these code words for 'come round to ours and do this'...'have this drug'...'chill-outs'...It all escalates.* (Aged 20-29)

The gay scene was described as 'fun', 'vibrant' and 'exciting', but also 'reckless', 'dangerous' and 'abusive'. Several men had experienced physical or sexual violence at saunas or nightclubs, sometimes associated with drug use, while cruising, or through meeting people via social media.



For peer review only - http://bmjopen.bmj.com/site/about/guidelines.xhtml

*Around that time there were two rapes on me...one happened in...a night club down the road...I went under on G [was under the influence of GHB/GBL]...and was attacked.* (Aged 20-29)

Several participants recalled changes in the gay scene, particularly the culture of drug use and sex. Changes were described in drug choices (e.g. mephedrone, GBL/GHB, crystal methamphetamine), context (chemsex), modes of administration (normalisation of injecting), and increasing availability of drugs at lower costs.

*Drugs have changed...there are more choices...GHB, mephedrone...which I was quite scared of in the beginning...but then it's normalised in the gay scene and you just tend to do what other people do. Same thing goes for injecting. I mean these days it's not seen as so scary.* (Aged 40-49)

A few other participants discussed the role of social norms and networks, as well as the pursuit of pleasure, as reasons for getting into chemsex:

*There's more drug taking in my social circle than I realised. I did occasionally take them [other recreational drugs], but it was the sex thing...I think it was discovering how good the sex was...on drugs in terms of the duration and intensity.* (Aged 50-59)

Chemsex enhanced and extended sex, reportedly impaired judgement, decreased risk perceptions, and moved boundaries.



For peer review only - http://bmjopen.bmj.com/site/about/guidelines.xhtml

For peer review only

> *I wasn't particularly stoned but you lose certain inhibitions and...I know there was a guy having sex with me, I was bottom, and I knew he had a condom to start off. But...I'm not quite sure he had a condom after.* (Aged 50-59)

***Community perceptions and experiences of HIV***

Respondents, particularly middle-aged or older men, described shifting perceptions about HIV. Some felt that the absence of stark media campaigns and deaths from AIDS associated with the 1980s-1990s had re-shaped attitudes towards HIV, reducing fears and risk perceptions. HIV was now frequently likened to having asthma or diabetes, was less concerning than hepatitis C, and was no longer viewed as a death sentence.

> *I think in London it's almost got to the point where people are not that concerned about it anymore. It's not looked at as a death sentence. I remember reading an article by a doctor, which I know a lot of gay people seem to have read...that he would rather have HIV than diabetes.* (Aged 30-39)

Living a normal, healthy life with HIV was often attributed to the availability of ART and good medical care. Believing that HIV is a manageable condition, reduced fears of HIV, and knowing healthy HIV-positive men led to complacency, with some men admitting their general denial or disregard of risks.

> *Everyone knows somebody positive now and knows that they're fit and healthy and they take a few pills a day...That's a huge factor in why so few people use protection anymore...because it has become a treatable illness...I think it changed everyone's risk calculations, because even if the worst did happen, it was no longer the worst.* (Aged 20-29)



For peer review only - http://bmjopen.bmj.com/site/about/guidelines.xhtml

Several participants said they balanced risks by testing regularly for HIV and other STIs, and/or that repeated negative results had encouraged further risk-taking.

> *I get into the way of thinking, 'oh it doesn't matter, you're going to die anyway one day...If you get sick you can take the pills' and it all becomes a bit blasé and a bit reckless. So it's taking a risk and then going to the clinic and finding out you're all clear.* (Aged 50-53)

Participants commonly associated an undetectable viral load, and occasionally ART, with lower risk of HIV transmission, a few specifically recalling scientific evidence. Some participants, typically younger men, made conscious choices to engage in condomless sex, including with HIV-positive (usually undetectable) men, influenced by their awareness of transmission risks and/or availability of ART, beliefs that HIV was no longer the worst outcome, or was inevitable considering the 'high prevalence of HIV in London'.

> *So we weren't always safe...I've read a lot...I think there was a French one [study] which was undetectable partner, negative partner, X number of however many tens of thousands of interactions, no transmissions. So, background reading kind of made me feel more comfortable with it [condomless sex with HIV-positive ex-partner].* (Aged 20-29)

Another participant, who said he had always had condomless sex, highlighted how sex with HIV-positive undetectable men was sometimes perceived as less risky than with HIV-negative men:

> *I was going to try and minimise the risks...and so I slept mostly with undetectable guys. I tried to avoid negative guys like the plague...Everyone told me, and what the research told me too, was that they were...the only ones who were going to possibly infect.* (Aged 20-29)



For peer review only - http://bmjopen.bmj.com/site/about/guidelines.xhtml

A few participants mentioned PrEP spontaneously and had asked for it at their sexual health clinics, but were not able to access it, while several had taken post-exposure prophylaxis (PEP) or discussed common perceptions of PEP as a backup option. Generally, many respondents thought the availability of ART, PrEP, PEP, or HIV tests, '*the undetectable factor*', changing discourses around HIV, or declining stigma had created a sense of apathy towards risks and susceptibility to HIV in the gay community. One man explained:

> *It's almost like they've been cured, 'Oh yeah, I'm HIV-positive but I'm undetectable'...which lessens the risk*. (Aged 20-29)

***Combined effects at the interface of individual and community levels on risk***

Our analysis revealed a layered complexity, with themes from both the individual and community/structural levels influencing participants' risk behaviours and risk of HIV infection. In this section we describe and give examples of combinations of factors from multiple levels that individuals attributed to their HIV infection, while also showing how the relative importance of contributory factors from individual and community/structural levels differed for each individual. We further illustrate how themes from each level could come together to heighten exposure to risk situations, for example, via the effects of impaired psychological wellbeing on sexual risk behaviour or elevated use of drugs and sexual networking apps, coupled with the lures of the social environment and exposure to high-risk contexts for sex. Changes in perceptions of risk and risk-benefit decisions around condomless sex were also brought about by individual-level psycho-social influences and experienced psychological trauma, which were compounded by changing community attitudes towards HIV and structural-level factors such as the availability of ART.



For peer review only - http://bmjopen.bmj.com/site/about/guidelines.xhtml

***Combinations of factors and perceived source of HIV infection***

A minority of participants thought they could pinpoint their source of HIV infection, although several indicated the point of infection was not important to them and mentioned they did not like to dwell on this or blame anyone. Most men thought they had contracted HIV from a casual sexual encounter at a club, sauna or sex party, or from someone they had met via social media. A few suspected their source of infection was a more regular partner, while a few cases of violence, for example sexual coercion or being purposefully overdosed by someone, were possibly related to HIV infection. Some participants discussed changes over time in their sexual risk-taking, for example increasing frequency of sex or beginning to have condomless sex, which were occasionally rationalised in terms of recent stressors. However, a greater number of men did not think their sexual behaviour had changed, and some were surprised they were not infected sooner.

Few participants thought that their HIV infection was the result of a single factor. More generally, participants thought that combinations of factors at both individual and community/structural levels contributed to risk behaviours and HIV infection. Within the combination of factors that contributed to HIV acquisition (Figure 1), the relative importance of factors from each level differed for each person.

A few participants specifically attributed their risk of HIV to early psycho-social issues as well as the social environment. For example, one participant felt his self-harming sexual behaviours stemmed from childhood and the violent relationship with his mother, but also highlighted the role of the "*abusive*" environment, including gay saunas, and normalisation of drug use:



For peer review only - http://bmjopen.bmj.com/site/about/guidelines.xhtml

> *I think with the sex, I think it's...environment, especially in South London. The increase of risk sex, chemsex, is becoming an epidemic, in my opinion. You hear of so many young gay men now who are positive...and through this lifestyle. It's very hedonistic, really nasty...I think, subsequently, living in South London has made me get HIV.* (Aged 20-29)

***Pathways to risk at the interface of individual and community levels***

Some explained how psycho-social issues resulted in their elevated use of drugs or sexual networking apps, which in turn widened their exposure to contexts for higher risk sex such as sex parties and chemsex.

> *I think the drugs make you want to do things like that [sex/ apps]. Being unhappy at work, the incredible amount of stress I went through, all of that combined made me feel a bit lonely as well...So I started to rely a bit on Grindr.* (Aged 30-39)

Psychological issues and drug use were often mentioned in combination. For example, one participant identified the important factors in his HIV infection as:

> *The drugs...but also depression because I didn't care about taking risks...I gave up*. (Aged 40-49)

Some participants who experienced psycho-social issues also identified ART and changing perceptions of HIV as factors that had consciously, or sub-consciously, influenced their risk behaviours. Risk-benefit decisions regarding sexual practices were altered, influencing decisions to engage in condomless sex, including with HIV-positive men, or imbuing a more general disregard of risks.



For peer review only - http://bmjopen.bmj.com/site/about/guidelines.xhtml

*When we were young adults, the fear of God was put into us. If you got it [HIV] you died...Now it is manageable...you could live a normal life...and you are thinking 'Okay, I live in a city with very high prevalence of this disease. I am at a crossroads in my life in terms of what I want to experience sexually'...I think the trauma I have gone through has changed what I perceive as real risk in life...There are far more important things...So it was a combination of all those different aspects, I had come to the conclusion that if I did become HIV-positive it wouldn't be a big event in my life.* (Aged 40-49)

The environment also enabled sexual exploration, particularly for those who had initially contained their sexuality, for example due to socio-cultural influences in their childhood. For a few participants, moving to London and fragmenting support networks had coincided with coming out, exposing them to new social and sexual norms including sex parties, group sex and chemsex.

*I was like a kid in a sweet shop. I wanted to experience everything, but I didn't necessarily have the rhetoric [support] around me to keep me safe.* (Aged 30-39)

***Relative importance of factors***

In contrast to the weight attributed to psycho-social factors by participants in the examples above, other participants thought that the social environment played a more substantial role than psychological factors in the build up to their diagnosis. For example, one man emphasised that recent stress, such as his father's ill-health, had less bearing on his "*dangerous lifestyle*" and HIV infection than the draws of the social scene:



For peer review only - http://bmjopen.bmj.com/site/about/guidelines.xhtml

*Probably they [stressors] were a lesser extent than 'I want to go out and have fun because I'm getting old and I'm not going to be able to do it in ten years' time', and that pull was probably stronger than any of the negative stuff about my life*. (Aged 50-59)

Some felt strongly that increasing use of sexual networking apps had played a major part in their own infection, through the ease of access to sexual partners, or in rising HIV infections in general, or that certain apps promoted promiscuity and irresponsibility. However, others re-iterated that it was difficult to distinguish the primary factor amidst a range of social environmental influences.

*The sex and the drugs and the apps all intertwined simultaneously and I can't really say which one led to the other.* (Aged 20-29)

Figure 2 gives worked examples of three contrasting cases, using the socio-ecological model, demonstrating the complexity of influences within and across multiple levels for each individual in terms of their risk of HIV acquisition. These examples also highlight the differences in relative importance of factors from each level, where for some men, individual-level psycho-social factors played a greater role in their infection compared to community or structural-level factors (e.g. cases A and B), while for others, community or structural-level factors contributed to a greater extent (e.g. case C). Within the individual level, there was typically a distinction in the balance of psycho-social influences towards those experienced early (e.g. case A) or more recently (e.g. case B), which in either case may accumulate to influence HIV risk.

For peer review only



For peer review only - http://bmjopen.bmj.com/site/about/guidelines.xhtml

## Discussion

This qualitative study among recently-infected MSM revealed a complex interplay of factors at the level of individuals and their community that influence risk behaviours and HIV acquisition. The relative importance of factors at each level varied for each person, with individual psycho-social factors enhancing vulnerability towards sexual risk situations, and features of the social risk environment and prevalent community beliefs encouraging risk-taking. A synergy of factors at the interface of these levels was ultimately important in affecting risk. Our findings reveal the broader context around new HIV infections in this population.

Psycho-social issues including early experiences of physical abuse, social rejection, isolation, drug use, anxiety or depression were common and heightened vulnerability to risk. Mental health conditions including major depression, anxiety disorders, suicidal tendencies, psychological distress and low self-esteem have been consistently reported at higher levels in MSM versus heterosexual men in high-income settings (21, 22). Several studies, predominantly American and including MSM populations, have also linked psycho-social issues such as depression, partner violence and childhood sexual abuse with condomless sex or risk of HIV acquisition (6, 23-26).

Notably, we found that a considerable number of participants had experienced multiple psycho-social issues over their life-course and/or a sudden series of traumatic events in the few years preceding their HIV diagnosis. These severely damaged their psychological well-being and appear important in placing some individuals at particularly high risk of engaging in unsafe sex or acquiring HIV. The magnified effect of experiencing multiple psycho-social issues is consistent with 'syndemic' theory, proposed initially to describe concurrent and mutually exacerbating epidemics, including poverty, racism, violence, substance use and HIV, in ethnic minority populations in the United States



For peer review only - http://bmjopen.bmj.com/site/about/guidelines.xhtml

of America (USA) (27). This concept was later extended to urban gay men, with depression, partner violence, suicidality, substance abuse and HIV identified as inter-related reinforcing epidemics (22, 25). Another USA-based longitudinal study has since provided compelling evidence that the accumulation of psycho-social conditions predicts HIV-related risk behaviours and HIV seroconversion among MSM (24).

Environments that normalise risk-taking, which in itself is a natural part of sexual discovery, can increase the likelihood of HIV acquisition, particularly for men whose individual-level experiences and other circumstances reduce their prioritising of sexual health. Chemsex was frequently reported in our sample and facilitated exposure to potential risks, for example through impaired judgement. This concurs with the findings of another qualitative study conducted in South London, which demonstrated that chemsex can carry more risk than drug-free sex, typically through longer duration and a greater number of partners (28). Underlying psycho-social factors, early-onset or more recent stressors, were occasionally related to engagement in chemsex in our study, although social norms sometimes appeared to be stronger influencing factors.

Sexual networking apps were commonly an accessory to drugs and sex, making it easy to meet partners and connect with networks where risk of HIV transmission might be higher. Our findings complement those of a recent cross-sectional study among young MSM in Britain and Ireland, suggesting that higher odds of high risk condomless sex were associated with, though not necessarily caused by, longer use of these technologies (13). However, our results highlight the difficulty in disentangling the role of sexual networking apps from other social environmental influences, including chemsex and other drug use, or underlying psycho-social influences.

Influenced by structural factors including the availability of ART, HIV was widely perceived among the men interviewed to be preventable and manageable, shaping attitudes to risk and behavioural



For peer review only - http://bmjopen.bmj.com/site/about/guidelines.xhtml

norms that, in turn, played a role in HIV acquisition. Some men had developed risk-reduction strategies accordingly, while reduced perceptions of risk also led more generally to complacency in terms of sexual practices. Findings to date on this topic have been mixed (29-33), with causality difficult to infer. However, our findings support those of more recent studies (10, 34), suggesting that optimism about HIV/treatment is playing an increasingly important role in sexual risk-taking.

The main strength of our study lies in the richness of data collected, covering a broad range of recurrent themes. Furthermore, interviews took place relatively recently after infection when recall was likely to be good. Nonetheless, details of participants' likely source of infection and related events may, naturally, be limited in accuracy. Many respondents disclosed sensitive personal issues, although we cannot rule out the possibility of social desirability bias. Participants were exclusively recruited from London/ Brighton and most were white, so the generalisability of findings to MSM in other areas, or to Black, Asian and other ethnic minorities (BAME), may be limited. However, we focussed on a key population of public health importance in the UK (2). It is also likely that our findings are applicable to MSM in other large cities, in the UK or similar international contexts, with sizeable MSM populations.

Our findings highlight the need for expanded access to packages of interventions that address exposures at multiple levels, if we are to limit new infections in this population. Examples already exist of multi-level or multi-faceted interventions (35) which, moving forwards, must be prioritised, particularly in the context of diminishing funding for sexual health and fragmentation of health service commissioning. Key components of a multi-level intervention would include, ideally, at the individual-level: improved clinical assessments to identify high-risk HIV-negative MSM who may benefit from enhanced support, including tailored support for young MSM, given the prominence of psycho-social issues, some of which emerged during adolescence; providing timely and flexible support, for example on-site in STI clinics and available immediately after testing, or via virtual web-



For peer review only - http://bmjopen.bmj.com/site/about/guidelines.xhtml

For peer review only

based technologies; and outreach activities to link vulnerable MSM to peer or community-based support groups, considering the deleterious effects of isolation and lack of social support. To address community-level risk factors, programmes should incorporate the provision of further information and education about chemsex, ideally including on-site advice in STI clinics. Training of health professionals on the social context around chemsex and sexual norms is essential, to enable culturally-competent discussions with MSM about their sexual behaviour, including engagement in chemsex, their risk-reduction strategies, and potential risks. In addition, sexual networking platforms are well-placed to deliver targeted interventions, including chemsex awareness and counselling options, while peer-led initiatives could assist in addressing complacency associated with shifting perceptions of HIV and ART, for example, by raising awareness of the social and psychological implications of an HIV diagnosis. At the structural level, our findings support policies that include access to PrEP, in combination with adherence support and promotion of frequent STI testing, for periods in which MSM find themselves at elevated risk due to compounded psycho-social life stressors.

In this paper we have shown that recently-acquired HIV infection among MSM reflects a complex web of factors operating at different levels. Individuals who experienced multiple stressors, gradually over the life-course or more suddenly, were especially vulnerable to HIV and being drawn into sexual risk situations, while the social environment created a context which enabled risk of HIV infection. While individuals were exposed to different sets of contributing circumstances, the interface of factors at each level was important in influencing risk behaviours and HIV acquisition. The circumstances surrounding HIV acquisition are complex and therefore require multi-level interventions that address individual and interpersonal psycho-social, community and structural level risk factors.



For peer review only - http://bmjopen.bmj.com/site/about/guidelines.xhtml

## Acknowledgements and funding

We would like to thank all the participants who took part in the interviews, as well as the clinic staff who assisted with recruitment. This report is independent research by the National Institute for Health Research. The research was funded by the National Institute for Health Research Health Protection Research Unit (NIHR HPRU) in Blood Borne and Sexually Transmitted Infections at University College London in partnership with Public Health England (PHE) and in collaboration with the London School of Hygiene and Tropical Medicine. The views expressed in this publication are those of the author(s) and not necessarily those of the NHS, the National Institute for Health Research, the Department of Health or Public Health England. Members of the Steering Committee of the NIHR HPRU: Caroline Sabin (Director), Anthony Nardone (PHE Lead), Catherine Mercer, Gwenda Hughes, Greta Rait, Jackie Cassell, William Rosenberg, Tim Rhodes, Kholoud Porter, and Samreen Ijaz.

## Potential conflicts of interest

JF has received grant support from Gilead Sciences and Merck. AG and KP have served on advisory boards for ViiV Healthcare. NN received honoraria for speaking and advising, travel grants and an institutional research grant from Bristol-Myers-Squibb, Janssen, ViiV Healthcare and Gilead Sciences. CO has received research grants, personal fees and non-financial support from Gilead Sciences, ViiV Healthcare, Bristol-Myers Squibb and MSD, and Janssen for advisory boards, lectureships and travel. All other authors declare that they have no conflict of interest.

## Author contributions

AG developed the discussion guide, scheduled and conducted the interviews, analysed the transcripts and wrote the manuscript. JF and KP conceived the study. JF recruited patients for interview, provided feedback on the discussion guide and early drafts of the manuscript. MG



For peer review only - http://bmjopen.bmj.com/site/about/guidelines.xhtml

provided social science advice regarding the analysis, interpretation and reporting of findings, double-coded a subset of the transcripts, and revised early drafts of the manuscript. SF, NN, AC, RG and CO organised and carried out recruitment of participants, and provided feedback on manuscript drafts. SC provided guidance on interpretation of preliminary findings and feedback on manuscript drafts. KP oversaw the development of fieldwork materials and early revisions of the manuscript. GH provided senior social science guidance on fieldwork, analysis, data interpretation and reporting, and revisions to early drafts of the manuscript. All authors contributed to and approved the current version of the manuscript.

## Data sharing statement

No additional data are available.

For peer review only

## References


1. Chau C, Kirwan P, Brown A, Gill N, Delpech V. HIV diagnoses, late diagnoses and numbers accessing treatment and care: 2016 report London: Public Health England; 2016 October 2016.
2. Skingsley A, Yin Z, Kirwan P, Croxford S, Chau C, Conti S, et al. HIV in the UK – Situation Report 2015: data to end 2014. London: Public Health England; 2015 November 2015.
3. Phillips AN, Cambiano V, Nakagawa F, Brown AE, Lampe F, Rodger A, et al. Increased HIV incidence in men who have sex with men despite high levels of ART-induced viral suppression: analysis of an extensively documented epidemic. PLoS One. 2013;8(2):e55312.
4. McCormack S, Dunn DT, Desai M, Dolling DI, Gafos M, Gilson R, et al. Pre-exposure prophylaxis to prevent the acquisition of HIV-1 infection (PROUD): effectiveness results from the pilot phase of a pragmatic open-label randomised trial. Lancet. 2016;387(10013):53-60.
5. Beyrer C, Baral SD, Collins C, Richardson ET, Sullivan PS, Sanchez J, et al. The global response to HIV in men who have sex with men. Lancet. 2016;388(10040):198-206.
6. Elam G, Macdonald N, Hickson FC, Imrie J, Power R, McGarrigle CA, et al. Risky sexual behaviour in context: qualitative results from an investigation into risk factors for seroconversion among gay men who test for HIV. Sex Transm Infect. 2008;84(6):473-7.
7. Macdonald N, Elam G, Hickson F, Imrie J, McGarrigle CA, Fenton KA, et al. Factors associated with HIV seroconversion in gay men in England at the start of the 21st century. Sex Transm Infect. 2008;84(1):8-13.
8. Desai M, Gill N, McCormack S, Dunn D, White E, Fox J, et al. Baseline predictors of HIV Infection in the no-PrEP group in the PROUD trial 22nd Annual Conference of the British HIV Association (BHIVA); 19-22 April 2016; Manchester2016.
9. Cohen MS, Chen YQ, McCauley M, Gamble T, Hosseinipour MC, Kumarasamy N, et al. Prevention of HIV-1 infection with early antiretroviral therapy. N Engl J Med. 2011;365(6):493-505.
10. Lampe F, Speakman A, Phillips A, Sherr L, Gilson R, Johnson M, et al. ART use, viral suppression, and sexual behaviour among HIV-diagnosed MSM in the UK: results from the Antiretrovirals, Sexual Transmission Risk and Attitudes (ASTRA) Study. Journal of the International AIDS Society. 2012;15((Suppl 4)):18143.
11. Gilbart VL, Simms I, Jenkins C, Furegato M, Gobin M, Oliver I, et al. Sex, drugs and smart phone applications: findings from semistructured interviews with men who have sex with men diagnosed with Shigella flexneri 3a in England and Wales. Sex Transm Infect. 2015;91(8):598-602.
12. Pufall E, Kall M, Shahmanesh M, Nardone A, Gilson R, Delpech V, et al. Chemsex and High-Risk Sexual Behaviours in HIV-Positive Men Who Have Sex With Men. Conference on Retroviruses and Opportunistic Infections; February 22–25 2016; Boston, Massachusetts2016.
13. Lorimer K, Flowers P, Davis M, Frankis J. Young men who have sex with men's use of social and sexual media and sex-risk associations: cross-sectional, online survey across four countries. Sex Transm Infect. 2016;92(5):371-6.
14. Dolling DI, Desai M, McOwan A, Gilson R, Clarke A, Fisher M, et al. An analysis of baseline data from the PROUD study: an open-label randomised trial of pre-exposure prophylaxis. Trials. 2016;17:163.
15. Baral S, Logie CH, Grosso A, Wirtz AL, Beyrer C. Modified social ecological model: a tool to guide the assessment of the risks and risk contexts of HIV epidemics. BMC Public Health. 2013;13:482.
16. Bronfenbrenner U. The ecology of human development: experiments by nature and design. . Cambridge, MA: Harvard University Press; 1979.
17. The UK register of HIV seroconverters: methods and analytical issues. UK register of HIV seroconverters (UKRHS) Steering Committee. Epidemiol Infect. 1996;117(2):305-12.




For peer review only - http://bmjopen.bmj.com/site/about/guidelines.xhtml

18. National Institute for Health Research. Health Protection Research Unit in Blood Borne and Sexually Transmitted Infections London: UCL Research Dept of Infection & Population Health; 2016 [Available from: http://bbsti.hpru.nihr.ac.uk/.
19. Sandelowski M. Whatever happened to qualitative description? Res Nurs Health. 2000;23(4):334-40.
20. Ritchie J, Lewis J. Qualitative research practice: a guide for social science students and researchers. London: Sage; 2003.
21. Sandfort TG, de Graaf R, Ten Have M, Ransome Y, Schnabel P. Same-sex sexuality and psychiatric disorders in the second Netherlands Mental Health Survey and Incidence Study (NEMESIS-2). LGBT Health. 2014;1(4):292-301.
22. Wolitski J, Stall RD, Valdiserri RO. Unequal Opportunity. Health Disparities Affecting Gay and Bisexual Men in the United States. New York: Oxford University Press; 2008.
23. Koblin BA, Husnik MJ, Colfax G, Huang Y, Madison M, Mayer K, et al. Risk factors for HIV infection among men who have sex with men. AIDS. 2006;20(5):731-9.
24. Mimiaga MJ, O'Cleirigh C, Biello KB, Robertson AM, Safren SA, Coates TJ, et al. The effect of psychosocial syndemic production on 4-year HIV incidence and risk behavior in a large cohort of sexually active men who have sex with men. J Acquir Immune Defic Syndr. 2015;68(3):329-36.
25. Mustanski B, Garofalo R, Herrick A, Donenberg G. Psychosocial health problems increase risk for HIV among urban young men who have sex with men: preliminary evidence of a syndemic in need of attention. Ann Behav Med. 2007;34(1):37-45.
26. DiClemente RJ, Crittenden CP, Rose E, Sales JM, Wingood GM, Crosby RA, et al. Psychosocial predictors of HIV-associated sexual behaviors and the efficacy of prevention interventions in adolescents at-risk for HIV infection: what works and what doesn't work? Psychosom Med. 2008;70(5):598-605.
27. Singer M. AIDS and the health crisis of the U.S. urban poor; the perspective of critical medical anthropology. Soc Sci Med. 1994;39(7):931-48.
28. Bourne A, Reid D, Hickson F, Torres-Rueda S, Weatherburn P. Illicit drug use in sexual settings ('chemsex') and HIV/STI transmission risk behaviour among gay men in South London: findings from a qualitative study. Sex Transm Infect. 2015;91(8):564-8.
29. Elford J. HIV treatment optimism and high-risk sexual behaviour among gay men: the attributable population risk. AIDS. 2004;18(16):2216-7.
30. Kalichman SC, Eaton L, Cain D, Cherry C, Fuhrel A, Kaufman M, et al. Changes in HIV treatment beliefs and sexual risk behaviors among gay and bisexual men, 1997-2005. Health Psychol. 2007;26(5):650-6.
31. Cox J, Beauchemin J, Allard R. HIV status of sexual partners is more important than antiretroviral treatment related perceptions for risk taking by HIV positive MSM in Montreal, Canada. Sex Transm Infect. 2004;80(6):518-23.
32. Vanable PA, Ostrow DG, McKirnan DJ. Viral load and HIV treatment attitudes as correlates of sexual risk behavior among HIV-positive gay men. J Psychosom Res. 2003;54(3):263-9.
33. Ostrow DE, Fox KJ, Chmiel JS, Silvestre A, Visscher BR, Vanable PA, et al. Attitudes towards highly active antiretroviral therapy are associated with sexual risk taking among HIV-infected and uninfected homosexual men. AIDS. 2002;16(5):775-80.
34. Brennan DJ, Welles SL, Miner MH, Ross MW, Rosser BR, Positive Connections T. HIV treatment optimism and unsafe anal intercourse among HIV-positive men who have sex with men: findings from the positive connections study. AIDS Educ Prev. 2010;22(2):126-37.
35. Chelsea and Westminster Hospital NHS Foundation Trust. Dean Street PRIME London: 56 Dean Street, Chelsea and Westminster Hospital NHS Foundation Trust,; 2016 [Available from: http://prime.dean.st/.



For peer review only - http://bmjopen.bmj.com/site/about/guidelines.xhtml

# Tables

**Table 1. Demographic characteristics of 21 participants**

| Characteristic | Category | Number of participants |
|---|---|---|
| Age (years) | 20-29 | 6 |
| | 30-39 | 6 |
| | 40-49 | 5 |
| | ≥50 | 4 |
| Ethnicity | White | 19 |
| | BAME | 2 |
| Education | Secondary | 9 |
| | Higher education | 12 |
| Employment | Employed | 18 |
| | Unemployed | 3 |

BAME Black, Asian and Minority Ethnic

For peer review only

# Figure legends

**Figure 1. Adapted socio-ecological framework of factors influencing risk of HIV acquisition among recently infected MSM in London and Brighton**

<<insert figure 1 here>>

ART anti-retroviral therapy; PEP post-exposure prophylaxis
This adapted socio-ecological framework (15, 16) depicts individual- and interpersonal-level psycho-social risk factors within the wider community and structural environments. Factors within and between each layer interact to influence risk behaviours and risk of HIV acquisition.

**Figure 2. Examples of combinations of factors, applying a socio-ecological framework, which contributed to risk behaviours and/or HIV acquisition for three participants**

<<insert figure 2 here>>

ART anti-retroviral therapy; IPV intimate partner violence
Individual and interpersonal psycho-social factors are depicted in the inner circle, factors at the community-level are shown in the middle circle, and factors at the structural-level are depicted in the outer circle. In all three cases, factors from all levels contributed to HIV risk, although their relative importance varied. Case A and B both experienced a high density of individual-level psycho-social factors, which played a greater role than community or structural-level factors. These two cases also illustrate a contrasting balance of factors within the individual-level, with Case A having experienced psycho-social factors that accumulated from a young age, and Case B having experienced only more recent stressors. In case C, individual-level psycho-social factors contributed to a lesser extent than community and structural-level factors.



For peer review only - http://bmjopen.bmj.com/site/about/guidelines.xhtml

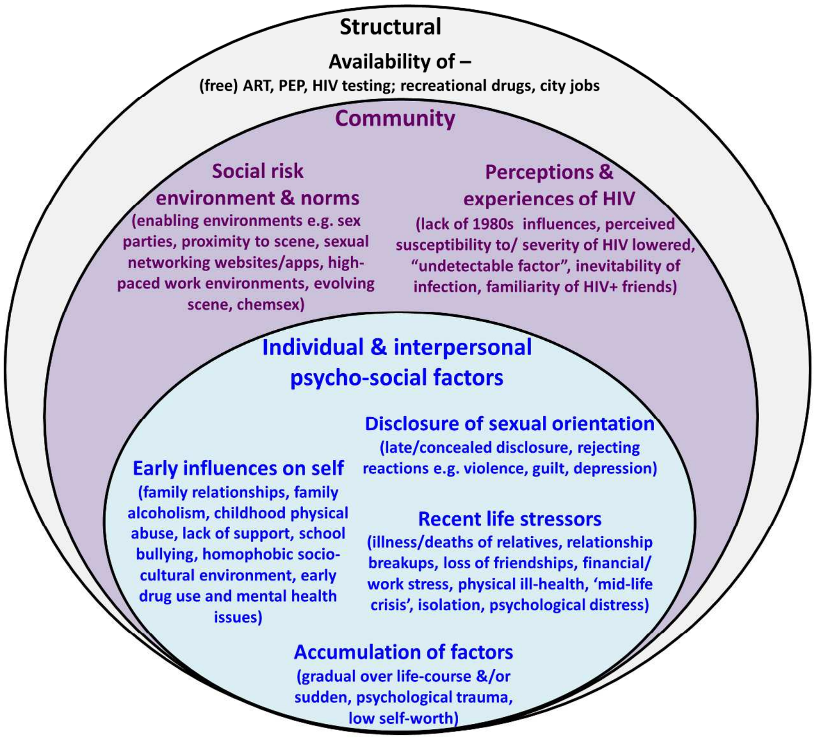


Figure 1. Adapted socio-ecological framework of factors influencing risk of HIV acquisition among recently infected MSM in London and Brighton

254x190mm (300 x 300 DPI)

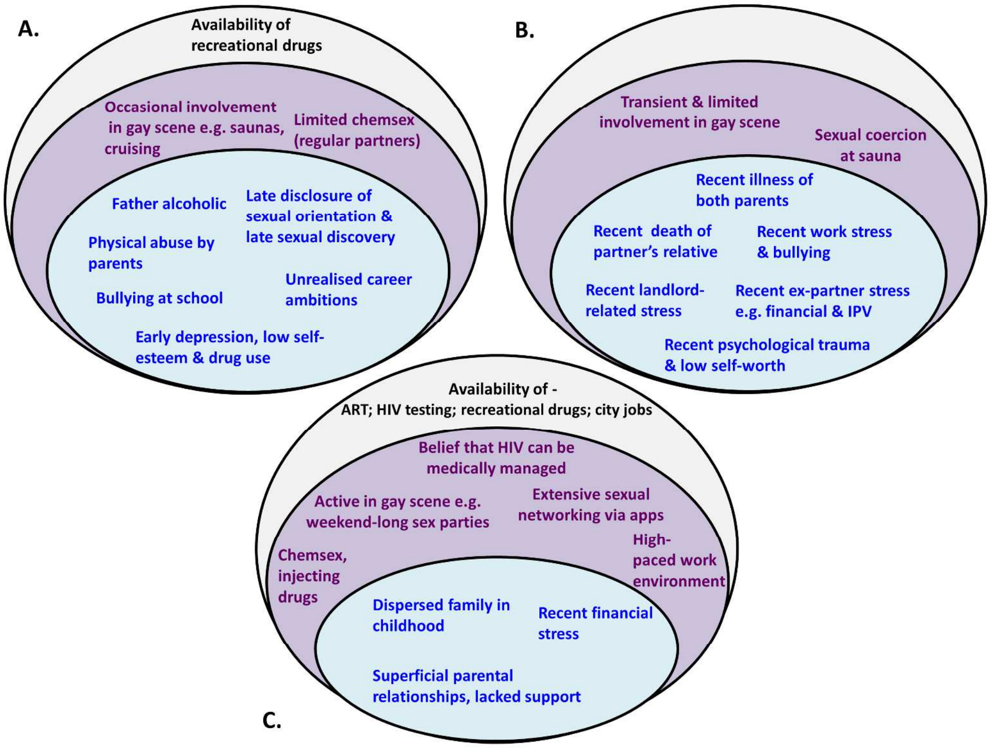


Figure 2. Examples of combinations of factors, applying a socio-ecological framework, which contributed to risk behaviours and/or HIV acquisition for three participants

254x190mm (300 x 300 DPI)

# Social factors related to HIV acquisition
# Interview discussion guide

## Introduction

- Thank for agreeing to participate
- Introduce self and organisation in collaboration with clinics
- Recap study objectives
- Remind participant they can stop the interview at any time or decline to answer
- Double check consent for recording the interview

## Ice-breaker & background

**Can you tell me a little bit about yourself and your background?**

- How old are you?
- Where are you from originally (born there)? A city or a rural area? Where do you live now? Who are you living with? How long have you been living there? If moved, when? From where? Why?
- Do you have family? Who? (Parents/ siblings – older or younger) Where are they living?
- What education have you received? University/higher education?
- Are you employed (or student)? What? Where? Since when?
- Are you in a relationship? Since when? Where is your partner from? If not in a relationship, previously in a relationship? When did this end? Relationships with men only, or men and women?
- Have you told anyone that you are gay or bisexual? When did you come out? How old were you then?

## Migration

**[If not originally from this city]**

**You said you moved to London/Brighton in yyyy. If you think back to that time, how were you feeling and how did you find this transition?**

- Moved alone or with someone else/ others?
- Sources of support?
- Any challenges?

For peer review only

## Circumstances around HIV infection

**Could you tell me about your life in the last year or so, just before you discovered you were HIV positive?**

- Monogamous/exclusive sexual relationship(s)?
- Or more casual sexual partners/ dating? Nature and number of partners? Preferred casual partners, or seeking long-term relationship?
- Where and how you met? Social activities? Clubs, bars, sex clubs, saunas, cruising (public environment)? Any change in habits?
- Recent travel (sex) abroad? (e.g. Amsterdam, Paris, Berlin)
- Internet, dating apps, other social media? Any change in use?
- Experienced sexual violence (in last year, or before, e.g. forced you to have sex)? Or other physical abuse (e.g. he hit you)? Shouting or threatening you (emotional abuse)? Any other frightening behaviour?
- Friends?
- How were you feeling emotionally (psychological well-being)? Self-confidence? What triggered any change?
- Health?
- Work?
- Financial situation?
- Home and family?
- Moving/re-location?
- Lifestyle?
- Drug or alcohol use? If yes, how did you get involved in/ what triggered this? Why?
- Any other challenges faced during this time?
- Negative reactions towards your sexuality/being gay? (Stigma (homophobia))

**Do you know when you might have caught HIV? How? From whom?**

- HIV-status and nature of sexual partner(s)?
- Sexual behaviour (condom use, breakage, receptive/insertive partner, ejaculation?)
- Were you (ever) forced into sex?
- To what extent did you see this [e.g. unprotected sex] as a risk to your sexual health? Why? (Risk perception and attitudes to unprotected sex)
- Did you try to manage this risk [e.g. sex without condom]? How? Any challenges? Ever discussed this with anyone – partners, friends, health or other support services? (Risk management)
- How were you feeling at this time?
- Drug (e.g. poppers, ecstasy) or alcohol use before or during sex?
- How did you (or other HIV-positive peers) become involved in [drug use/ high risk practices/ gay social scene]?
- What (other) factors do you think contributed (to risk behaviour and getting infected)? (Probe for social factors above) How?
- Why do you think you got HIV *now*? What factors or events triggered this?

For peer review only

## Overcoming challenges and staying HIV-negative

**You mentioned […] were difficulties you faced at this time. How might any of these difficulties/events have been overcome?**

- Support services? What exactly? By whom? How would this have helped?

**What would have helped you to stay HIV negative?**

## Previous STI/HIV testing patterns and counselling

**Can you tell me about any contact you had with health services for STI testing *before* you were diagnosed with HIV?**

- How many times did you get tested for HIV?
- How did you react to these (negative) results? Effect on sexual behaviour?
- Diagnosed with other STIs? Which? When?
- Experiences with using these services?
- Perceptions of the counselling given (including drug use counselling). What advice given? Helpful or not? How easy to understand the advice or put it into practice? Timing of advice (started receiving in time/ too late?)
- Any other advice or support (clinic-based or other, e.g. social groups, internet-based) you received, or wish you had received?

## Initial perceptions of HIV care and support services

**Some people can find it difficult to continue attending clinics or to take treatment in the long-term. Based on your initial experiences of the HIV services you have received and your personal life at the moment, do you anticipate any difficulties with being able to continue attending?**

- What difficulties?
- Disclosure (planned/already? How did that go?) Experienced/anticipated negative reactions (stigma)?
- Probe for any of the social or psychological factors discussed above
- Attended/ experiences or perceptions of other non-clinic support services?
- What would help you to continue attending/ follow clinical advice?

## END OF INTERVIEW

- Thank participant for their time
- Reassure confidentiality of the discussion
- Provide information on relevant support services

For peer review only

Table 1

**Standards for Reporting Qualitative Research (SRQR)[a]**

| No. | Topic | | Item |
|---|---|---|---|
| | **Title and abstract** | | |
| S1 | Title | page 1 | Concise description of the nature and topic of the study Identifying the study as qualitative or indicating the approach (e.g., ethnography, grounded theory) or data collection methods (e.g., interview, focus group) is recommended |
| S2 | Abstract | page 2 | Summary of key elements of the study using the abstract format of the intended publication; typically includes background, purpose, methods, results, and conclusions |
| | **Introduction** | page 4-5 | |
| S3 | Problem formulation | | Description and significance of the problem/phenomenon studied; review of relevant theory and empirical work; problem statement |
| S4 | Purpose or research question | | Purpose of the study and specific objectives or questions |
| | **Methods** | page 6-8 | |
| S5 | Qualitative approach and research paradigm | p7 | Qualitative approach (e.g., ethnography, grounded theory, case study, phenomenology, narrative research) and guiding theory if appropriate; identifying the research paradigm (e.g., postpositivist, constructivist/ interpretivist) is also recommended; rationale[b] |
| S6 | Researcher characteristics and reflexivity | p7 p26 | Researchers' characteristics that may influence the research, including personal attributes, qualifications/experience, relationship with participants, assumptions, and/or presuppositions; potential or actual interaction between researchers' characteristics and the research questions, approach, methods, results, and/or transferability |
| S7 | Context | p6 | Setting/site and salient contextual factors; rationale[b] |
| S8 | Sampling strategy | p6 | How and why research participants, documents, or events were selected; criteria for deciding when no further sampling was necessary (e.g., sampling saturation); rationale[b] |
| S9 | Ethical issues pertaining to human subjects | p8 | Documentation of approval by an appropriate ethics review board and participant consent, or explanation for lack thereof; other confidentiality and data security issues |
| S10 | Data collection methods | p7 | Types of data collected; details of data collection procedures including (as appropriate) start and stop dates of data collection and analysis, iterative process, triangulation of sources/methods, and modification of procedures in response to evolving study findings; rationale[b] |
| S11 | Data collection instruments and technologies | p7 | Description of instruments (e.g., interview guides, questionnaires) and devices (e.g., audio recorders) used for data collection; if/how the instrument(s) changed over the course of the study |
| S12 | Units of study | p6-7 p9 | Number and relevant characteristics of participants, documents, or events included in the study; level of participation (could be reported in results) |
| S13 | Data processing | p7-8 | Methods for processing data prior to and during analysis, including transcription, data entry, data management and security, verification of data integrity, data coding, and anonymization/deidentification of excerpts |
| S14 | Data analysis | p7-8 | Process by which inferences, themes, etc., were identified and developed, including the researchers involved in data analysis; usually references a specific paradigm or approach; rationale[b] |
| S15 | Techniques to enhance trustworthiness | p7-8 | Techniques to enhance trustworthiness and credibility of data analysis (e.g., member checking, audit trail, triangulation); rationale[b] |
| | **Results/findings** | | |
| S16 | Synthesis and interpretation | p9-23 | Main findings (e.g., interpretations, inferences, and themes); might include development of a theory or model, or integration with prior research or theory |
| S17 | Links to empirical data | p9-23 | Evidence (e.g., quotes, field notes, text excerpts, photographs) to substantiate analytic findings |
| | **Discussion** | | |
| S18 | Integration with prior work, implications, transferability, and contribution(s) to the field | p24-27 | Short summary of main findings; explanation of how findings and conclusions connect to, support, elaborate on, or challenge conclusions of earlier scholarship; discussion of scope of application/ generalizability; identification of unique contribution(s) to scholarship in a discipline or field |
| S19 | Limitations | p26 | Trustworthiness and limitations of findings |

(*Table continues*)



168x233mm (300 x 300 DPI)

Table 1
(Continued)

| No. | Topic | | Item | |
|---|---|---|---|---|
| | **Other** | | | |
| S20 | Conflicts of interest | p28 | Potential sources of influence or perceived influence on study conduct and conclusions; how these were managed | ✓ |
| S21 | Funding | p 28 | Sources of funding and other support; role of funders in data collection, interpretation, and reporting | ✓ |

[a]The authors created the SRQR by searching the literature to identify guidelines, reporting standards, and critical appraisal criteria for qualitative research; reviewing the reference lists of retrieved sources; and contacting experts to gain feedback. The SRQR aims to improve the transparency of all aspects of qualitative research by providing clear standards for reporting qualitative research.
[b]The rationale should briefly discuss the justification for choosing that theory, approach, method, or technique rather than other options available, the assumptions and limitations implicit in those choices, and how those choices influence study conclusions and transferability. As appropriate, the rationale for several items might be discussed together.

should provide evidence (e.g., examples, quotes, or text excerpts) to substantiate the main analytic findings.[20,29]

**Discussion.** The discussion of qualitative results will generally include connections to existing literature and/or theoretical or conceptual frameworks, the scope and boundaries of the results (transferability), and study limitations.[10–12,28] In some qualitative traditions, the results and discussion may not have distinct boundaries; we recommend that authors include the substance of each item regardless of the section in which it appears.

## Discussion

The purpose of the SRQR is to improve the quality of reporting of qualitative research studies. We hope that these 21 recommended reporting standards will assist authors during manuscript preparation, editors and reviewers in evaluating a manuscript for potential publication, and readers when critically appraising, applying, and synthesizing study findings. As with other reporting guidelines,[35–37] we anticipate that the SRQR will evolve as it is applied and evaluated in practice. We welcome suggestions for refinement.

Qualitative studies explore "how?" and "why?" questions related to social or human problems or phenomena.[10,38] Purposes of qualitative studies include understanding meaning from participants' perspectives (How do they interpret or make sense of an event, situation, or action?); understanding the nature and influence of the context surrounding events or actions; generating theories about new or poorly understood events, situations, or actions; and understanding the processes that led to a desired (or undesired) outcome.[38] Many different approaches (e.g., ethnography, phenomenology, discourse analysis, case study, grounded theory) and methodologies (e.g., interviews, focus groups, observation, analysis of documents) may be used in qualitative research, each with its own assumptions and traditions.[1,2] A strength of many qualitative approaches and methodologies is the opportunity for flexibility and adaptability throughout the data collection and analysis process. We endeavored to maintain that flexibility by intentionally defining items to avoid favoring one approach or method over others. As such, we trust that the SRQR will support all approaches and methods of qualitative research by making reports more explicit and transparent, while still allowing investigators the flexibility to use the study design and reporting format most appropriate to their study. It may be helpful, in the future, to develop approach-specific extensions of the SRQR, as has been done for guidelines in quantitative research (e.g., the CONSORT extensions).[37]

### Limitations, strengths, and boundaries

We deliberately avoided recommendations that define methodological rigor, and therefore it would be inappropriate to use the SRQR to judge the quality of research methods and findings. Many of the original sources from which we derived the SRQR were intended as criteria for methodological rigor or critical appraisal rather than reporting; for these, we inferred the information that would be needed to evaluate the criterion. Occasionally, we found conflicting recommendations in the literature (e.g., recommending specific techniques such as multiple coders or member checking to demonstrate trustworthiness); we resolved these conflicting recommendations through selection of the most frequent recommendations and by consensus among ourselves.

Some qualitative researchers have described the limitations of checklists as a means to improve methodological rigor.[13] We nonetheless believe that a checklist for reporting standards will help to enhance the transparency of qualitative research studies and thereby advance the field.[29,39]

Strengths of this work include the grounding in previously published criteria, the diversity of experience and perspectives among us, and critical review by experts in three countries.

### Implications and application

Similar to other reporting guidelines,[35–37] the SRQR may be viewed as a starting point for defining reporting standards in qualitative research. Although our personal experience lies in health professions education, the SRQR is based on sources originating in diverse health care and non-health-care fields. We intentionally crafted the SRQR to include various paradigms, approaches, and methodologies used in qualitative research. The elaborations offered in



164x233mm (300 x 300 DPI)

For peer review only - http://bmjopen.bmj.com/site/about/guidelines.xhtml
